# Supplementary material for: MAAMOUL: metabolic network-based discovery of microbiome-metabolome shifts in disease
Source: Bioinformatics. 2026 Aug 21;42(Suppl 2):btag467. doi: 10.1093/bioinformatics/btag467 (PMC13501328; doi:10.1093/bioinformatics/btag467)
Supplement: Supplementary file 1 [file btag467_supplementary_data.pdf]

Supplementary data

Supplementary Note 1: IBS-associated microbiome-metabolome modules ... *2*
Supplementary Methods ... *3*
Construction of the global metabolic network ... 3
Microbiome-metabolome datasets acquisition and processing ... 3
Differential abundance analysis ... 4
MAAMOUL implementation, parameters and sensitivity analysis ... 4
Identification of overlaps between modules and KEGG pathways ... 5
Pathway-based analysis ... 5
Visualizations ... 5
Supplementary Figures ... *6*
Supplementary Figure S1: A bipartite global metabolic network ... 6
Supplementary Figure S2: Distribution of p-values from differential abundance analysis and the corresponding fitted mixture models ... 7
Supplementary Figure S3: Sensitivity analysis – Cumulative size of output modules using different pipeline parameters ... 8
Supplementary Figure S4: Microbiome-metabolome modules associated with Crohn's disease ... 9
Supplementary Figure S5: Microbiome functional shifts associated with IBS ... 10
Supplementary Tables ... *11*
Supplementary Table S1: Description of studies included in the analysis ... 11
Supplementary Table S2: Differential abundance analysis statistics per dataset ... 11
Supplementary Table S3: Beta-uniform mixture model parameters per dataset ... 12
Supplementary Table S4: Metabolic network statistics per dataset ... 12
Supplementary Table S5: Modules overview per dataset ... 13
Supplementary Table S6: Detailed modules per dataset ... 16
Supplementary Table S7: Modules significant overlaps with KEGG pathways ... 24
Supplementary Table S8: Pathway-disease associations (KEGG pathways) using multiple analysis approaches ... 27
References ... *29*

Supplementary Note 1: IBS-associated microbiome-metabolome modules

As mentioned in the main text, we utilized MAAMOUL to investigate the metabolic shifts associated with Irritable Bowel Syndrome (IBS). IBS is a highly heterogeneous and multifactorial condition with multiple disease subtypes and clinical manifestations. Although several studies have reported microbiome taxonomic and functional associations with IBS symptom severity, or comorbidities[1–3], these signatures are generally weaker than those observed in other gastrointestinal diseases, and underlying mechanisms remain largely unclear. Applying MAAMOUL to microbiome-metabolome data from Jeffery et al.[4] (with 78 IBS and 58 control samples), we found 13 IBS-associated modules (FDR<0.1), of which, two (Modules #2 and #4) were supported by both metabolite and metagenomic EC features (Supplementary Figure 5).

Module #2 (Supplementary Figure 5A) includes 4 amino acids increased in IBS, namely lysine, glutamine, serine and tryptophan (Trp), alongside a decrease in arginine and multiple genes involved in amino acid biosynthesis and utilization. This pattern aligns with reports of elevated proteolytic activity in the IBS gut, where bacteria increasingly bypass carbohydrate fermentation in favor of protein breakdown[5,6]. Arginine depletion may reflect increased microbial utilization, consistent with enrichment of arginase and arginine decarboxylase genes, and potentially linked to the expansion of *Enterobacteriaceae* that utilize a "linear arginine pathway" to outcompete commensals[7]. Similarly, the enrichment of Trp and Trp-related genes (e.g., tryptophanase and tryptophan synthase) aligns well with previous implications of dysregulated Trp metabolism in IBS pathogenesis and symptoms[8,9], potentially via microbial modulation of Trp flux and activation of the host's kynurenine pathway, which in turn influence intestinal motility and visceral sensitivity in IBS[10].

Module #4 (Supplementary Figure 5B), in contrast, captures a different shift involving purine and nicotinate metabolism. It includes decreased levels of adenosine, adenine, and nicotinamide alongside increased abundance of multiple ECs overlapping the purine and nicotinate/nicotinamide pathways (Supplementary Table S7). Disturbances in purine metabolism have recently been proposed as a novel mechanism underlying IBS[11], potentially reflecting elevated microbial utilization of purine nucleotides that may contribute to epithelial metabolic stress and impaired mucosal repair. Consistent with this hypothesis, the module contains xanthine dehydrogenase genes (EC 1.17.1.4), previously reported to be elevated in IBS patients with predominant constipation[12]. The module also includes multiple enzymes at the intersection of purine metabolism and nicotinate/nicotinamide metabolism (e.g., EC 2.4.2.1), as well as nicotinamidase (EC 3.5.1.19), consistent with reduced fecal nicotinamide (a form of vitamin B3 that enters the gut lumen through circulating host nicotinamide[13]) and increased microbial conversion of nicotinamide to nicotinate in IBS[14].

In summary, these results demonstrate MAAMOUL's ability to identify mechanistically coherent, multi-omic functional units even within the heterogeneous landscape of IBS.

Supplementary Methods

Construction of the global metabolic network

We constructed a metabolic network of enzyme nodes (represented by EC codes) and metabolite nodes (represented by KEGG compound ID's), using data from the KEGG database (FTP 2021-05-10 release)[15]. Notably, in contrast to the common metabolic network representation, where nodes represent metabolites and metabolic reactions are denoted by hyperedges, our representation includes metabolic reactions (coded by corresponding EC numbers) as nodes as well, resulting in a bipartite graph with two types of nodes (ECs and metabolites) and edges connecting EC nodes to their substrate and product metabolite nodes.

The network was constructed as follows: First, KEGG reaction ID's were mapped to their main substrates and products (given by compound ID's) using the file "reaction_mapformula.lst" from the "ligand" subdirectory. Then, each reaction was mapped to its corresponding EC (one or more) using the file "reaction_enzyme.list". Metabolite and enzyme names, as well as other properties, were extracted from the "compound" and "enzyme" files, respectively. The unique list of EC-metabolite pairs was then used as edges in the bipartite network. This preliminary global metabolic network included 3,793 enzyme nodes, 4,516 metabolite nodes, and 12,241 edges.

We further pre-processed the network by performing the following steps: Nodes with a rank (number of neighbors in the network) > 20 were removed from the network alongside all their connected edges. Overall, 27 metabolites were removed in this step (including, for example, Acetyl-CoA, ammonia, glucose and CO2), and 24 EC nodes. This removal of "currency" nodes (mostly metabolites) is a common practice in metabolic modeling[16], resulting in a network with a more informative topology. Singleton nodes were also removed. After this step, the network included 3,752 EC nodes, 4,321 metabolite nodes, and 10,697 edges. Next, EC nodes that were not linked in KEGG to any bacterial organism, as determined using the KEGG files "enzyme" ("ligand" subdirectory) and "taxonomic_rank" ("genes" subdirectory), were omitted from the network, as well as metabolite nodes that became singletons, resulting in 2,348 EC nodes and 2,861 metabolite nodes remaining in the graph. Lastly, connected components in the graph that included less than 10 nodes were removed. We refer to the resulting network, after the preprocessing steps described above, as the "global metabolic network", containing 2,172 EC nodes, 2,539 metabolite nodes and 6,253 edges, grouped into 13 connected components, the largest of which including 4,439 nodes. Supplementary Figure S1 presents node rank distributions before and after network processing, and a visualization of the final graph.

Microbiome-metabolome datasets acquisition and processing

In this work, we used data from the following studies: Franzosa et al.[17], Jeffery et al.[18] and Wang et al.[19]. For each cohort we obtained: (a) Metagenomics-based enzyme profiles; (b) Metabolite profiles as published or shared by the authors of the original studies; (c) Subject/sample metadata including demographics, disease state (healthy/disease), etc. One exception was the data from Jeffery et al., for which instead of metabolite profiles (that were unavailable), we obtained p-values per metabolite based on differential abundance testing from the original publication.

Enzyme profiles, given by Enzyme Commission (EC) numbers, were computed using HUMANn3[20], with default parameters, after quality filtering using the 'fastp' tool (version 0.23)[21], and host-reads removal using bowtie2 and

the GRCh38 human reference genome (https://www.ncbi.nlm.nih.gov/datasets/genome/GCF_000001405.26), with the "sensitive" flag. As some of the EC codes obtained by HUMANn3 have been officially deprecated, we mapped deprecated EC numbers to their updated ones using the ENZYME database from the Expasy resource (https://ftp.expasy.org/databases/enzyme/enzyme.dat). The resulting EC profiles were normalized using MUSiCC[22]. Finally, rare EC's, defined as those observed in less than 10% of the samples were removed.

Metabolites were mapped, where possible, to their KEGG compound identifiers, either by the authors of the original studies or using the id conversion utility of MetaboAnalyst[23]. Metabolites that could not be mapped into KEGG identifiers were discarded. Rare metabolites, appearing in less than 10% of samples, were removed as well. Missing metabolite values were imputed using half the minimal observed value of each metabolite. Lastly, metabolite values were log-transformed to account for heteroscedasticity[24]. Further details and processing notes for each dataset, as well as accession codes for obtaining the raw data, are provided in Supplementary Table S1.

Differential abundance analysis

For each dataset, we ran differential abundance testing for each EC/metabolite feature using linear mixed models, as implemented in the "MaAsLin2" R package (version 1.8)[25]. Briefly, in each model, the EC/metabolite feature was the dependent variable, the study group (i.e. case/control label) was an independent (predictor) variable, and additional covariates/confounders, as controlled for in the original studies, were added as additional covariates. The p-value of the "study-group" variable coefficient was then recorded. The list of covariates used per dataset, the number of features (after pre-processing) that underwent testing, and additional statistics are given in Supplementary Table S2.

Notably, by using only p-values (rather than effect sizes), we focused solely on the significance of each feature's association with disease, ignoring the direction of association (i.e. whether the feature's abundance increased or decreased in disease). Our motivation for this choice was to relax the assumption that a functional module perturbed in disease is consistently upregulated or consistently downregulated, and rather allow identification of any module demonstrating shifts in activity in disease states. Similar methods for finding disease-associated modules in biological networks have adopted the same heuristic[26,27]. The method could, however, easily be adapted to find only consistent up-/down-regulated modules using one-sided statistical tests.

MAAMOUL implementation, parameters and sensitivity analysis

The MAAMOUL method is implemented in R (version 4.1). We use the "fitBumModel" function from the "BioNet" R package (version 1.54)[28] to obtain maximum-likelihood estimations for mixture model parameters, and the R package "igraph" (version 1.3.4)[29] for miscellaneous graph-related computations.

The method requires three main parameters: (a) An FDR threshold, determining the threshold used for defining which nodes will be considered as module "anchors" (default: 0.1); (b) k, determining a maximal distance between nodes for them to be considered as being part of the same disease-associated module (default: 4); and (c) The height at which we "cut" the tree obtained by hierarchical clustering of all anchor nodes (default: 0.8).

To assess how different parameter settings impact the identified modules, we ran MAAMOUL with several different combinations of parameter values (FDR threshold parameter, and k parameter; see Supplementary Figure S3). As expected, less stringent FDR thresholds, and higher levels of k, both resulted in an overall increase in the

number of nodes included in the obtained modules. Importantly, however, across all datasets, and across all parameter settings, the cumulative size of all obtained modules was significantly higher compared to modules identified in node-permuted graphs. Overall, different settings may produce more/ fewer modules, of increased/decreased size, and parameter choice should be made based on data characteristics and desired sensitivity. The parameters chosen for the analysis presented (which are the default parameters described above) were selected to maintain a balance between discovery and stringency.

Identification of overlaps between modules and KEGG pathways

For each MAAMOUL-identified module (complete modules, not only anchor nodes), we identified all the KEGG-pathways that it significantly overlapped. To this end, we created a table mapping each node in the global metabolic network (either EC or metabolite) to all pathways in which they appear in KEGG, using the tables “enzyme_pathway.list” and “compound_pathway.list” from the “ligand” KEGG directory. Per module, we identified all pathways for which an overlap of at least 3 nodes existed, and then used a hypergeometric test to obtain an “enrichment p-value”. P-values were then FDR-corrected. All overlaps with an FDR < 0.1 are listed in Supplementary Table S7, and visualized in Figures 3B and S4B.

Pathway-based analysis

To compare the custom modules identified by MAAMOUL to the common practice of pathway-based analysis, we implemented an Over Representation Analysis (ORA). Specifically, we used the same categorization of EC and metabolite features into significantly disease-associated (i.e., “anchors” in MAAMOUL’s terminology) and non-disease-associated. We then used a hypergeometric test to examine, for each pathway, whether it was significantly enriched with disease associated ECs/metabolites. For consistency, for each KEGG pathway we used only the nodes that were also included in our global network (removing, for example, currency metabolites).

For metagenomic data only, we also report results from a standard differential abundance test, but applied at the whole pathway level. Specifically, the abundance of each pathway was computed as the sum of the relative abundances of all the ECs in that pathway, where the abundance of an EC that was included in multiple pathways is evenly distributed among them (the “uniform fractional mapping” approach[30]). We then used a linear mixed model (as described above for the individual features) to determine whether the abundance of each pathway was significantly different in cases versus controls. All results from these pathway-level analyses are provided in Supplementary Table S8.

Visualizations

Network visualizations were created using the R package “igraph” (version 1.3.4)[29], the RCy3 package (version 2.26.0)[31], and the Cytoscape software (version 3.10.2)[32].

Supplementary Figures

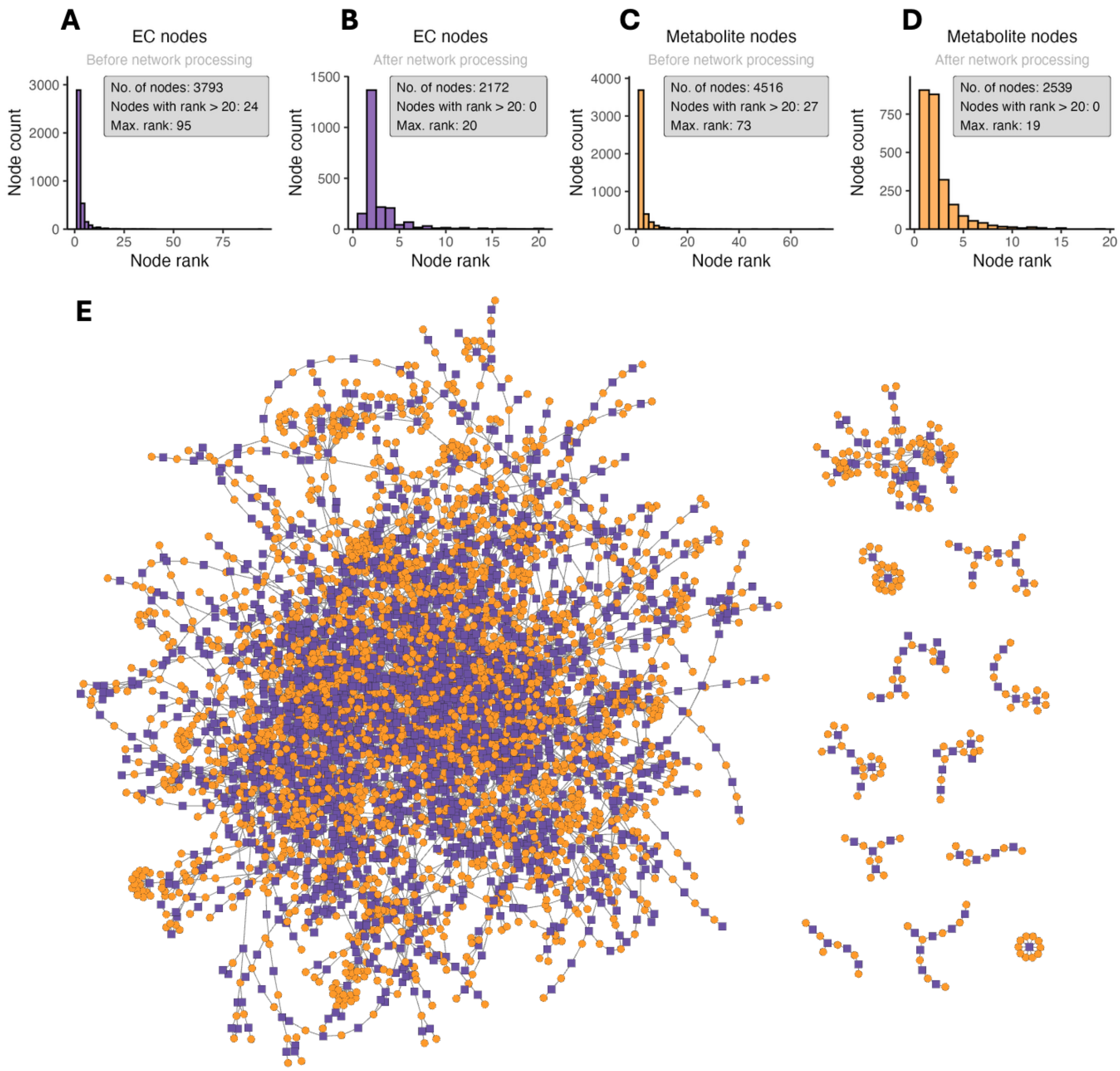

Supplementary Figure S1: A bipartite global metabolic network

(A-D) The distribution of node degrees (ranks) in the global bipartite network. Left panels (A,C) describe EC nodes, right panels (B, D) describe metabolite nodes, upper panels (A, B) describe the full network before any processing, and the lower panels (C, D) describe the final network after various processing steps; **(E)** A cytoscape visualization of the entire bipartite global metabolic network after processing.

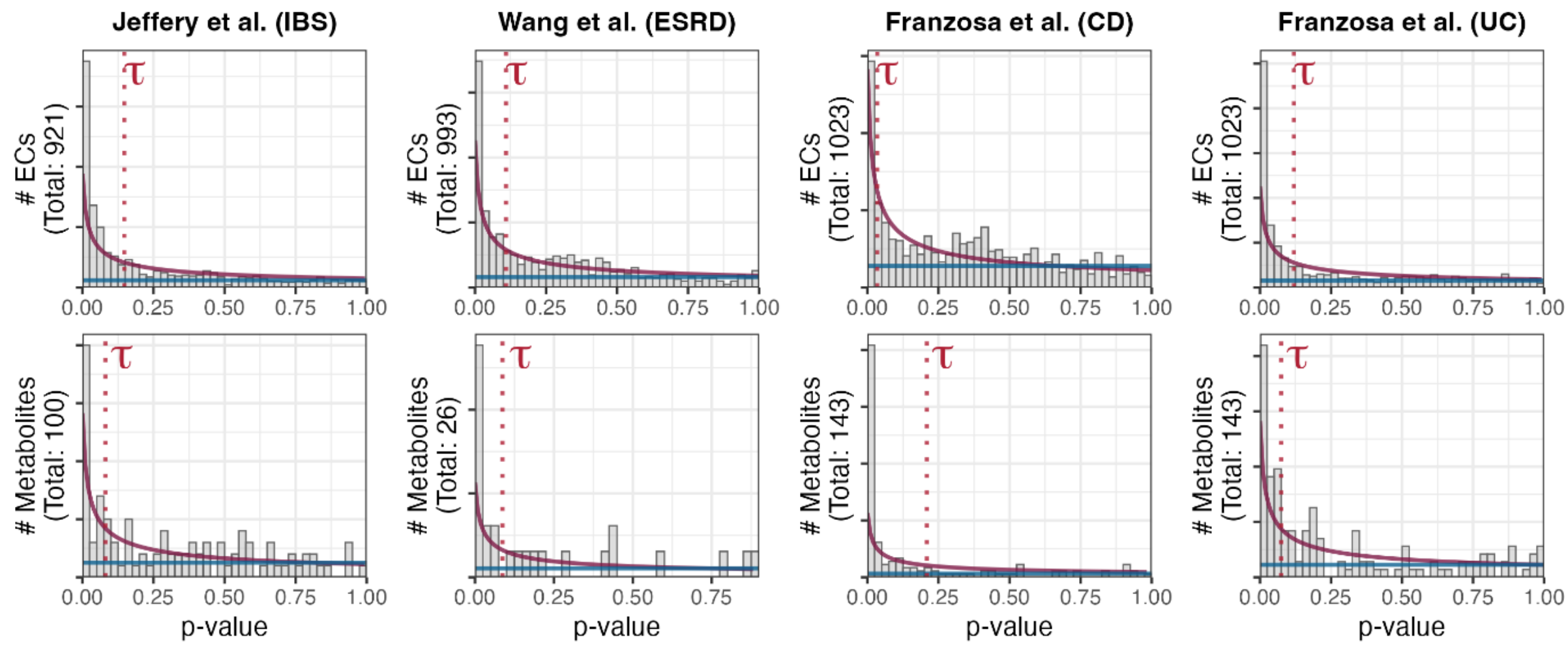

Supplementary Figure S2: Distribution of p-values from differential abundance analysis and the corresponding fitted mixture models

For each dataset, the distribution of p-values from the differential abundance testing (Methods) is presented, for EC features (upper panels) and metabolite features (lower panels) separately. Purple and blue overlaying lines illustrate the fitted beta distribution and uniform distribution, respectively, of the beta-uniform mixture (BUM) models. Red dashed line represent the selected p-value cutoff for defining "anchor" nodes, τ, signifying a false discovery rate of 0.1 and using the formulation by Pounds and Morris[33].

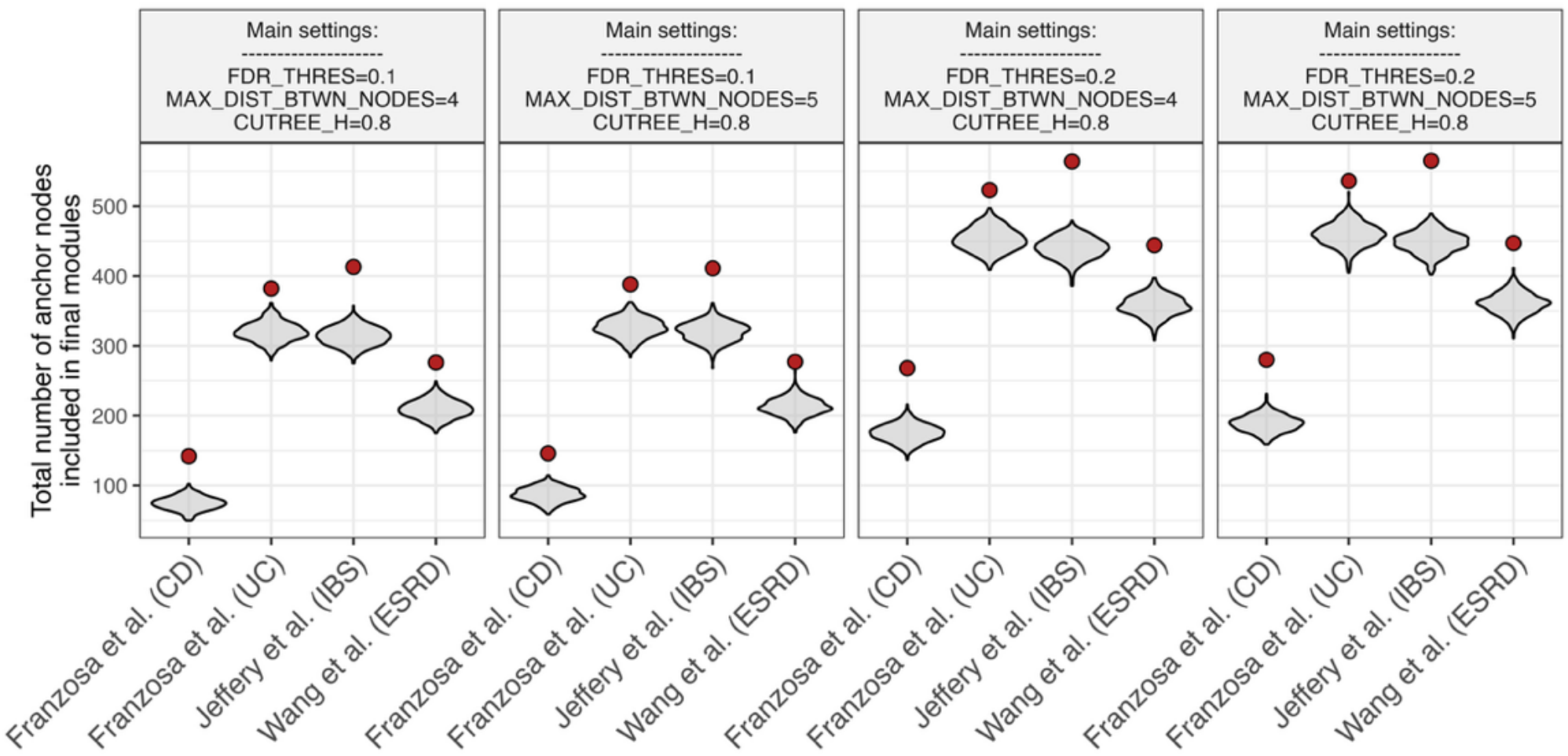

Supplementary Figure S3: Sensitivity analysis – Cumulative size of output modules using different pipeline parameters

Each panel represents a different set of pipeline parameters. See Methods for parameter descriptions. For each dataset (x axis), the red point represents the cumulative number of nodes included in the identified modules (regardless of the modules' statistical significance as determined by the topology-aware permutation test). Grey violins represent the distribution of cumulative numbers of nodes for node-permuted networks, as a null distribution.

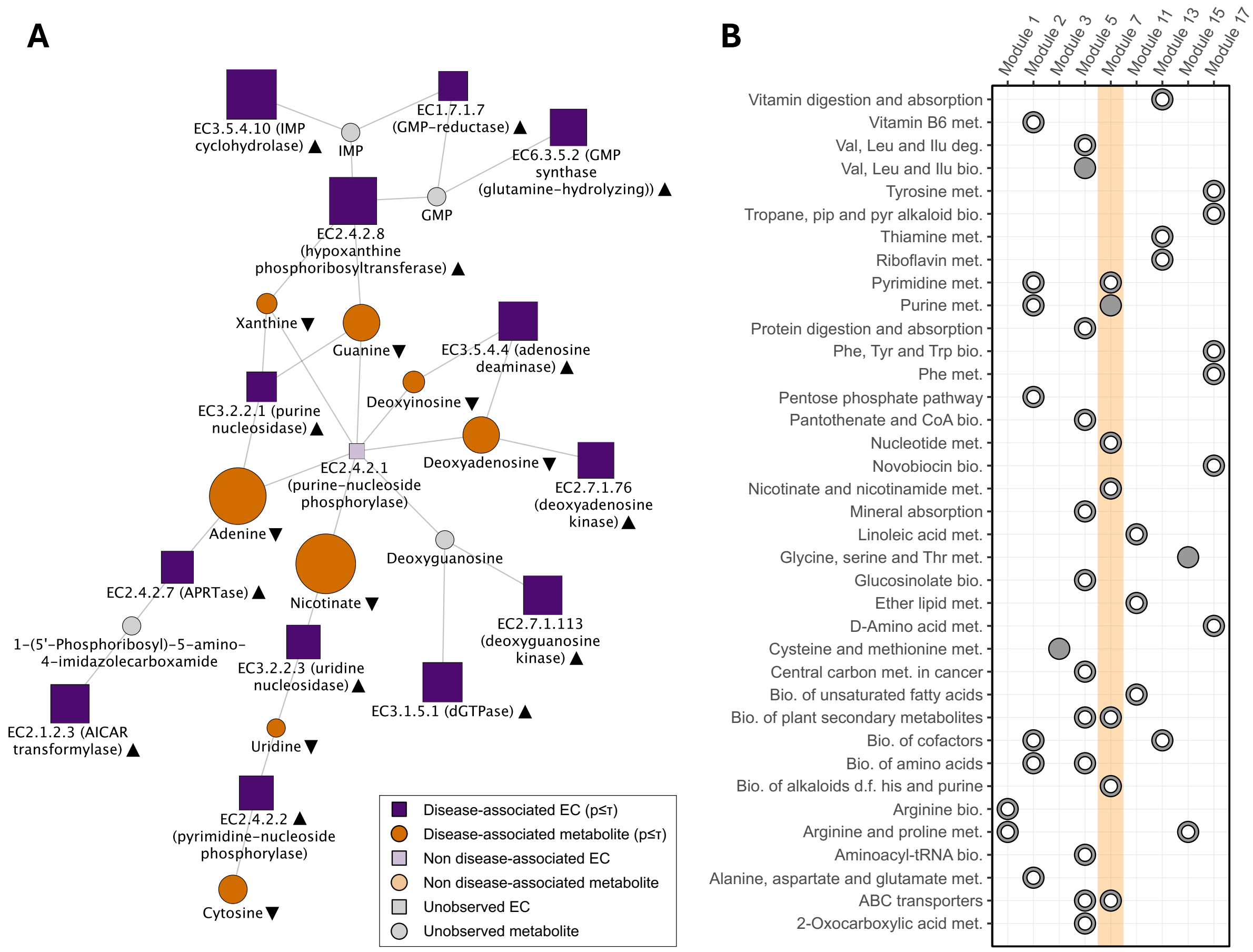

Supplementary Figure S4: Microbiome-metabolome modules associated with Crohn's disease

(A) An illustration of Module #7 identified by MAAMOUL using data from Crohn's disease (CD) patients and healthy controls from Franzosa et al.[17]. (B) Significant overlaps between CD-associated modules identified by MAAMOUL (columns) and pre-defined KEGG metabolic pathways (rows). Overlaps with Module #7 presented in panel (A) are highlighted. See legend of Figure 3.

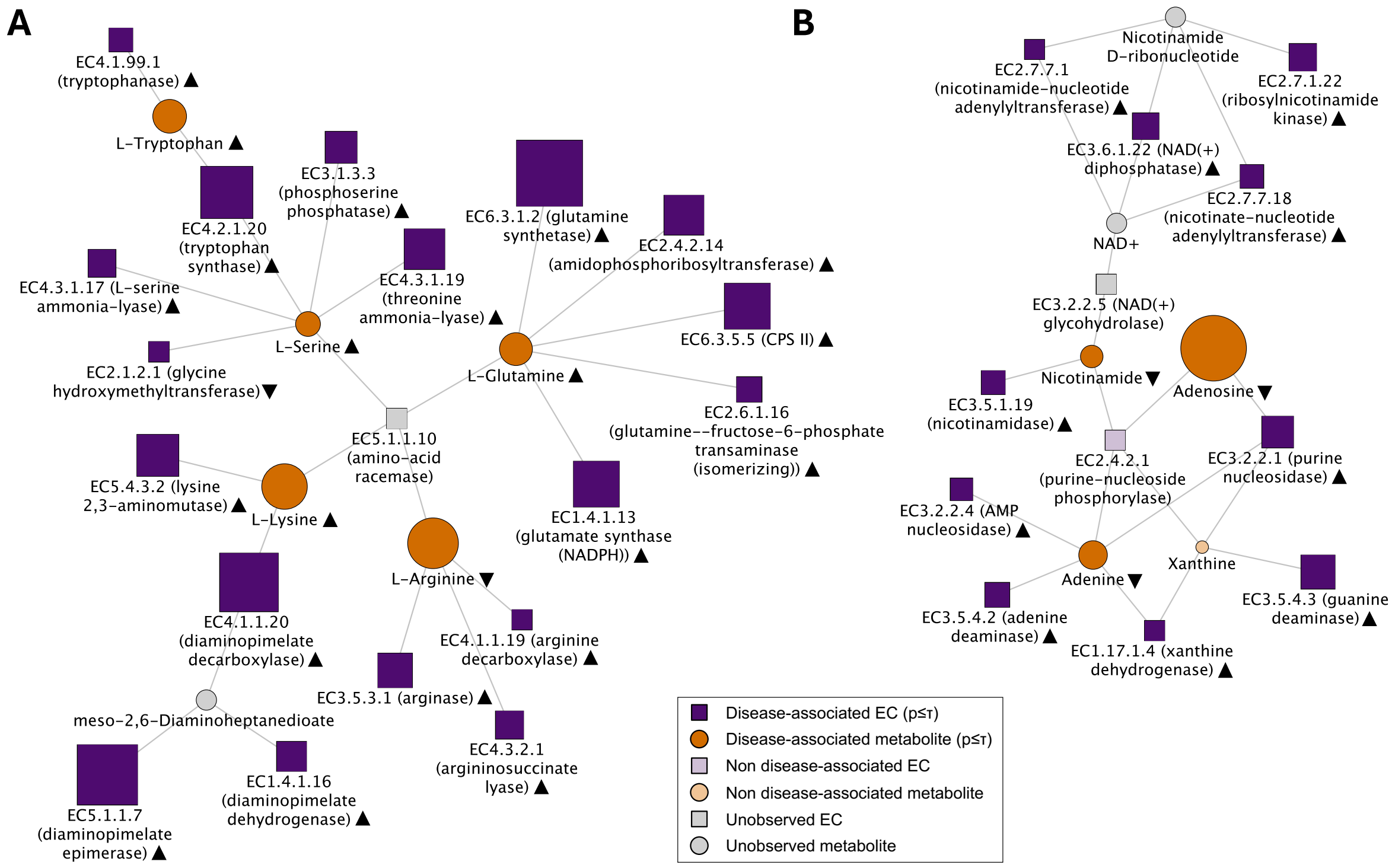

Supplementary Figure S50: Microbiome functional shifts associated with IBS

An illustration of Module #2 **(A)**, and Module #4 **(B)** identified by MAAMOUL applied to the IBS dataset from Jeffery et al.[18]. See legend of Figure 3.

Supplementary Tables

Supplementary Table S1: Description of studies included in the analysis

Dataset	Cohort description	No. samples		Microbiome data	Metabolome data		Metadata location	Publication	Comments
		Control	Case	Raw data location	Instrument	Data location			
Franzosa et al. (CD)	Inflammatory bowel disease patients and controls (PRISM cohort). - CD and healthy only.	56	73	BioProject: PRJNA400072 / Paper SI	Four complimentary LC-MS methods	Paper SI	Paper SI	DOI: 10.1038/s41564-018-0306-4	The dataset includes two different cohorts: the main PRISM cohort (from Boston, USA) and a validation cohort from the Netherlands.
Franzosa et al. (UC)	Inflammatory bowel disease patients and controls (PRISM cohort). - UC and healthy only.	56	70	BioProject: PRJNA400072 / Paper SI	Four complimentary LC-MS methods	Paper SI	Paper SI	DOI: 10.1038/s41564-018-0306-4	The dataset includes two different cohorts: the main PRISM cohort (from Boston, USA) and a validation cohort from the Netherlands.
Wang et al. (ESRD)	Adults with end-stage renal disease (ESRD) and controls.	67	220	BioProject: PRJNA449784	GC-MS	MetaboLights database: accession number MTBLS700	MetaboLights database: accession number MTBLS700	DOI: 10.1136/gutjnl-2019-319766	
Jeffery et al. (IBS)	Adults with and without IBS (Rome IV criteria)	58	78	BioProject: PRJEB42304	LC-MS	Paper SI	Shared by authors	DOI: 10.1053/j.gastro.2019.11.301	Metabolite levels were unavailable. Instead, pre-computed differential abundance p-values were obtained from supplementary tables.

Supplementary Table S2: Differential abundance analysis statistics per dataset

Dataset	Fixed effects for DA analysis	Feature type	No. of samples	No. of features	No. of p-values < 0.05	No. of FDR values < 0.05	No. of p-values < 0.1	No. of FDR values < 0.1
Wang et al. (ESRD)	Age, Gender, BMI	ECs	287	993	289	87	388	123
Wang et al. (ESRD)	Age, Gender, BMI	Metabolites	287	26	11	6	13	7
Franzosa et al. (CD)	Age, immunosuppressant, mesalamine, steroids, Batch	ECs	129	1023	206	19	281	34
Franzosa et al. (CD)	Age, immunosuppressant, mesalamine, steroids, Batch	Metabolites	129	143	84	48	93	66
Franzosa et al. (UC)	Age, immunosuppressant, mesalamine, steroids, Batch	ECs	126	1023	412	6	513	41
Franzosa et al. (UC)	Age, immunosuppressant, mesalamine, steroids, Batch	Metabolites	126	143	43	12	63	19
Jeffery et al. (IBS)		ECs	136	921	353	190	462	291
Jeffery et al. (IBS)		Metabolites	139	100	23	15	35	23

* Note 1: Numbers include all observed features in the data, after pre-processing (e.g. removal of rare features). Some of these features may have been missing from the global network (see Table S4).
* Note 2: Significance thresholds in this table are based on standard FDR thresholds, and not on the mixture models (see Table S3).

Supplementary Table S3: Beta-uniform mixture model parameters per dataset.

Dataset	Feature type	BUM parameter: a (shape)	BUM parameter: λ (mixture)	Pipeline parameter: FDR threshold	Final p-value threshold based on BUM	Percent of features below p-value threshold ("anchors")
Jeffery et al. (IBS)	EC	0.3579	0	0.1	0.1372	54.23
Jeffery et al. (IBS)	Metabolite	0.2719	0.3156	0.1	0.0709	34.8
Wang et al. (ESRD)	EC	0.4002	0	0.1	0.0991	38.74
Wang et al. (ESRD)	Metabolite	0.1527	0.4463	0.1	0.077	38.04
Franzosa et al. (CD)	EC	0.5161	0.086	0.1	0.0246	14.22
Franzosa et al. (CD)	Metabolite	0.1955	0.1597	0.1	0.1989	65.52
Franzosa et al. (UC)	EC	0.3876	0	0.1	0.1094	50.13
Franzosa et al. (UC)	Metabolite	0.4507	0	0.1	0.0645	31.61

* BUM = Beta-uniform mixture model

Supplementary Table S4: Metabolic network statistics per dataset

Dataset	No. of metabolite nodes with a p-value	No. of EC nodes with a p-value	No. of 'anchor' metabolite nodes	No. of 'anchor' EC nodes	No. of metabolite nodes in global network	No. of EC nodes in global network
Wang et al. (ESRD)	26	977	16	547	2539	2172
Franzosa et al. (CD)	99	1004	66	255	2539	2172
Franzosa et al. (UC)	99	1004	31	499	2539	2172
Jeffery et al. (IBS)	98	904	29	393	2539	2172

Supplementary Table S5: Modules overview per dataset

Dataset	Module ID	Module p-value (network permutation test)	Module FDR (network permutation test)	No. of DA nodes in module	No. of DA metabolites in module	No. of DA ECs in module	Full module size
Franzosa et al. (CD)	1	0.036	**0.0780**	5	2	3	8
Franzosa et al. (CD)	2	0.004	**0.0347**	11	2	9	14
Franzosa et al. (CD)	3	0.006	**0.0390**	4	3	1	7
Franzosa et al. (CD)	4	0.064	0.1040	8	3	5	11
Franzosa et al. (CD)	5	0.026	**0.0662**	5	4	1	7
Franzosa et al. (CD)	6	0.17	0.2009	4	2	2	7
Franzosa et al. (CD)	7	0.002	**0.0347**	21	8	13	26
Franzosa et al. (CD)	8	0.302	0.3141	3	1	2	5
Franzosa et al. (CD)	9	0.004	**0.0347**	3	3	0	5
Franzosa et al. (CD)	10	0.274	0.2968	3	1	2	6
Franzosa et al. (CD)	11	0.012	**0.0446**	4	3	1	6
Franzosa et al. (CD)	12	0.028	**0.0662**	5	5	0	6
Franzosa et al. (CD)	13	0.01	**0.0433**	6	3	3	8
Franzosa et al. (CD)	14	0.06	0.1040	5	1	4	9
Franzosa et al. (CD)	15	0.028	**0.0662**	3	2	1	5
Franzosa et al. (CD)	16	0.092	0.1259	4	1	3	5
Franzosa et al. (CD)	17	0.042	**0.0840**	7	1	6	11
Franzosa et al. (CD)	18	0.214	0.2419	4	1	3	5
Franzosa et al. (CD)	19	0.078	0.1193	4	0	4	6
Franzosa et al. (CD)	20	0.136	0.1684	3	0	3	6
Franzosa et al. (CD)	21	0.018	**0.0585**	11	0	11	18
Franzosa et al. (CD)	22	0.008	**0.0416**	6	0	6	10
Franzosa et al. (CD)	23	0.086	0.1242	3	0	3	7
Franzosa et al. (CD)	24	0.346	0.3460	3	0	3	5
Franzosa et al. (CD)	25	0.124	0.1612	4	0	4	7
Franzosa et al. (CD)	26	0.046	**0.0854**	3	0	3	4
Franzosa et al. (UC)	1	0.008	**0.0596**	5	1	4	8
Franzosa et al. (UC)	2	0.002	**0.0223**	17	2	15	21
Franzosa et al. (UC)	3	0.07	0.2315	5	1	4	8
Franzosa et al. (UC)	4	0.28	0.3633	3	1	2	5
Franzosa et al. (UC)	5	0.002	**0.0223**	11	2	9	11
Franzosa et al. (UC)	6	0.002	**0.0223**	23	5	18	30
Franzosa et al. (UC)	7	0.282	0.3633	4	1	3	5
Franzosa et al. (UC)	8	0.02	**0.0957**	6	2	4	7
Franzosa et al. (UC)	9	0.352	0.4288	3	1	2	5
Franzosa et al. (UC)	10	0.156	0.2773	11	1	10	16
Franzosa et al. (UC)	11	0.34	0.4219	4	1	3	5
Franzosa et al. (UC)	12	0.526	0.5594	3	1	2	5
Franzosa et al. (UC)	13	0.484	0.5230	3	1	2	3
Franzosa et al. (UC)	14	0.01	**0.0670**	10	1	9	14
Franzosa et al. (UC)	15	0.124	0.2773	5	0	5	7
Franzosa et al. (UC)	16	0.038	0.1675	4	0	4	6
Franzosa et al. (UC)	17	0.208	0.2959	3	0	3	6
Franzosa et al. (UC)	18	0.002	**0.0223**	9	0	9	13
Franzosa et al. (UC)	19	0.436	0.4869	3	0	3	5
Franzosa et al. (UC)	20	0.368	0.4326	5	0	5	9
Franzosa et al. (UC)	21	0.658	0.6580	3	0	3	5
Franzosa et al. (UC)	22	0.156	0.2773	4	0	4	5
Franzosa et al. (UC)	23	0.002	**0.0223**	9	0	9	12
Franzosa et al. (UC)	24	0.014	**0.0782**	10	0	10	15
Franzosa et al. (UC)	25	0.25	0.3418	3	0	3	5
Franzosa et al. (UC)	26	0.194	0.2954	12	0	12	16
Franzosa et al. (UC)	27	0.074	0.2315	13	0	13	17
Franzosa et al. (UC)	28	0.124	0.2773	3	0	3	4
Franzosa et al. (UC)	29	0.148	0.2773	4	0	4	6
Franzosa et al. (UC)	30	0.154	0.2773	4	0	4	7
Franzosa et al. (UC)	31	0.388	0.4482	3	0	3	4
Franzosa et al. (UC)	32	0.076	0.2315	23	0	23	30
Franzosa et al. (UC)	33	0.59	0.5989	3	0	3	5
Franzosa et al. (UC)	34	0.168	0.2773	3	0	3	5
Franzosa et al. (UC)	35	0.36	0.4307	4	0	4	5
Franzosa et al. (UC)	36	0.202	0.2959	7	0	7	9
Franzosa et al. (UC)	37	0.118	0.2773	5	0	5	7
Franzosa et al. (UC)	38	0.176	0.2773	3	0	3	5
Franzosa et al. (UC)	39	0.06	0.2233	7	0	7	12
Franzosa et al. (UC)	40	0.456	0.5009	3	0	3	5
Franzosa et al. (UC)	41	0.002	**0.0223**	9	0	9	13
Franzosa et al. (UC)	42	0.11	0.2773	4	0	4	7
Franzosa et al. (UC)	43	0.148	0.2773	3	0	3	5
Franzosa et al. (UC)	44	0.258	0.3457	3	0	3	6
Franzosa et al. (UC)	45	0.014	**0.0782**	4	0	4	7
Franzosa et al. (UC)	46	0.004	**0.0383**	8	0	8	12
Franzosa et al. (UC)	47	0.16	0.2773	4	0	4	5
Franzosa et al. (UC)	48	0.006	**0.0503**	7	0	7	10
Franzosa et al. (UC)	49	0.04	0.1675	4	0	4	6
Franzosa et al. (UC)	50	0.172	0.2773	4	0	4	7
Franzosa et al. (UC)	51	0.05	0.1971	7	0	7	10
Franzosa et al. (UC)	52	0.17	0.2773	4	0	4	6
Franzosa et al. (UC)	53	0.17	0.2773	3	0	3	4
Franzosa et al. (UC)	54	0.308	0.3894	3	0	3	4
Franzosa et al. (UC)	55	0.178	0.2773	5	0	5	7
Franzosa et al. (UC)	56	0.56	0.5863	5	0	5	8
Franzosa et al. (UC)	57	0.426	0.4838	3	0	3	5
Franzosa et al. (UC)	58	0.212	0.2959	5	0	5	7
Franzosa et al. (UC)	59	0.148	0.2773	5	0	5	8
Franzosa et al. (UC)	60	0.17	0.2773	3	0	3	5
Franzosa et al. (UC)	61	0.02	**0.0957**	4	0	4	6
Franzosa et al. (UC)	62	0.13	0.2773	5	0	5	8

Franzosa et al. (UC)	63	0.072	0.2315	3	0	3	4
Franzosa et al. (UC)	64	0.156	0.2773	4	0	4	6
Franzosa et al. (UC)	65	0.1	0.2773	4	0	4	6
Franzosa et al. (UC)	66	0.21	0.2959	3	0	3	4
Franzosa et al. (UC)	67	0.59	0.5989	3	0	3	4
Jeffery et al. (IBS)	1	0.254	0.3651	5	1	4	7
Jeffery et al. (IBS)	2	0.002	**0.0345**	23	5	18	25
Jeffery et al. (IBS)	3	0.048	0.1274	12	1	11	15
Jeffery et al. (IBS)	4	0.002	**0.0345**	13	3	10	18
Jeffery et al. (IBS)	5	0.512	0.5791	8	1	7	11
Jeffery et al. (IBS)	6	0.1	0.2156	4	2	2	6
Jeffery et al. (IBS)	7	0.314	0.4167	5	1	4	5
Jeffery et al. (IBS)	8	0.792	0.8280	3	1	2	3
Jeffery et al. (IBS)	9	0.194	0.3187	7	1	6	9
Jeffery et al. (IBS)	10	0.642	0.7031	4	1	3	5
Jeffery et al. (IBS)	11	0.394	0.4904	4	1	3	7
Jeffery et al. (IBS)	12	0.832	0.8568	5	1	4	7
Jeffery et al. (IBS)	13	0.094	0.2137	6	0	6	9
Jeffery et al. (IBS)	14	0.246	0.3651	3	0	3	5
Jeffery et al. (IBS)	15	0.032	0.1227	26	0	26	36
Jeffery et al. (IBS)	16	0.01	**0.0767**	8	0	8	12
Jeffery et al. (IBS)	17	0.464	0.5336	6	0	6	9
Jeffery et al. (IBS)	18	0.042	0.1270	4	0	4	7
Jeffery et al. (IBS)	19	0.046	0.1270	9	0	9	15
Jeffery et al. (IBS)	20	0.096	0.2137	16	0	16	22
Jeffery et al. (IBS)	21	0.038	0.1270	11	0	11	16
Jeffery et al. (IBS)	22	0.406	0.4915	9	0	9	12
Jeffery et al. (IBS)	23	0.046	0.1270	7	0	7	11
Jeffery et al. (IBS)	24	0.082	0.1951	3	0	3	5
Jeffery et al. (IBS)	25	0.172	0.3036	3	0	3	5
Jeffery et al. (IBS)	26	0.614	0.6833	5	0	5	6
Jeffery et al. (IBS)	27	0.022	0.1084	6	0	6	10
Jeffery et al. (IBS)	28	0.424	0.5044	4	0	4	6
Jeffery et al. (IBS)	29	0.062	0.1584	8	0	8	12
Jeffery et al. (IBS)	30	0.254	0.3651	4	0	4	7
Jeffery et al. (IBS)	31	0.024	0.1104	5	0	5	8
Jeffery et al. (IBS)	32	0.042	0.1270	4	0	4	6
Jeffery et al. (IBS)	33	0.018	**0.0955**	10	0	10	16
Jeffery et al. (IBS)	34	0.174	0.3036	3	0	3	4
Jeffery et al. (IBS)	35	0.858	0.8706	5	0	5	9
Jeffery et al. (IBS)	36	0.272	0.3809	3	0	3	5
Jeffery et al. (IBS)	37	0.908	0.9080	7	0	7	11
Jeffery et al. (IBS)	38	0.006	**0.0518**	9	0	9	15
Jeffery et al. (IBS)	39	0.276	0.3809	3	0	3	5
Jeffery et al. (IBS)	40	0.014	**0.0878**	3	0	3	5
Jeffery et al. (IBS)	41	0.16	0.3036	3	0	3	4
Jeffery et al. (IBS)	42	0.248	0.3651	3	0	3	5
Jeffery et al. (IBS)	43	0.328	0.4270	4	0	4	6
Jeffery et al. (IBS)	44	0.104	0.2175	4	0	4	7
Jeffery et al. (IBS)	45	0.398	0.4904	4	0	4	7
Jeffery et al. (IBS)	46	0.778	0.8259	5	0	5	7
Jeffery et al. (IBS)	47	0.002	**0.0345**	3	0	3	5
Jeffery et al. (IBS)	48	0.014	**0.0878**	3	0	3	5
Jeffery et al. (IBS)	49	0.306	0.4140	5	0	5	7
Jeffery et al. (IBS)	50	0.176	0.3036	7	0	7	11
Jeffery et al. (IBS)	51	0.016	**0.0920**	4	0	4	7
Jeffery et al. (IBS)	52	0.174	0.3036	3	0	3	5
Jeffery et al. (IBS)	53	0.004	**0.0460**	18	0	18	25
Jeffery et al. (IBS)	54	0.368	0.4702	4	0	4	6
Jeffery et al. (IBS)	55	0.03	0.1218	7	0	7	9
Jeffery et al. (IBS)	56	0.144	0.2839	3	0	3	5
Jeffery et al. (IBS)	57	0.67	0.7223	6	0	6	9
Jeffery et al. (IBS)	58	0.204	0.3199	3	0	3	5
Jeffery et al. (IBS)	59	0.006	**0.0518**	4	0	4	5
Jeffery et al. (IBS)	60	0.002	**0.0345**	4	0	4	8
Jeffery et al. (IBS)	61	0.204	0.3199	5	0	5	7
Jeffery et al. (IBS)	62	0.08	0.1951	3	0	3	5
Jeffery et al. (IBS)	63	0.044	0.1270	4	0	4	6
Jeffery et al. (IBS)	64	0.44	0.5146	6	0	6	10
Jeffery et al. (IBS)	65	0.114	0.2314	3	0	3	5
Jeffery et al. (IBS)	66	0.034	0.1235	3	0	3	4
Jeffery et al. (IBS)	67	0.19	0.3187	3	0	3	6
Jeffery et al. (IBS)	68	0.004	**0.0460**	3	0	3	5
Jeffery et al. (IBS)	69	0.026	0.1121	3	0	3	5
Wang et al. (ESRD)	1	0.154	0.1953	3	2	1	4
Wang et al. (ESRD)	2	0.516	0.5160	3	1	2	4
Wang et al. (ESRD)	3	0.128	0.1707	7	1	6	10
Wang et al. (ESRD)	4	0.38	0.3875	7	1	6	10
Wang et al. (ESRD)	5	0.122	0.1669	5	1	4	8
Wang et al. (ESRD)	6	0.004	**0.0130**	4	0	4	6
Wang et al. (ESRD)	7	0.002	**0.0087**	5	0	5	8
Wang et al. (ESRD)	8	0.036	**0.0693**	4	0	4	6
Wang et al. (ESRD)	9	0.004	**0.0130**	5	0	5	8
Wang et al. (ESRD)	10	0.002	**0.0087**	4	0	4	6
Wang et al. (ESRD)	11	0.012	**0.0328**	5	0	5	8
Wang et al. (ESRD)	12	0.002	**0.0087**	6	0	6	9
Wang et al. (ESRD)	13	0.25	0.2674	3	0	3	5
Wang et al. (ESRD)	14	0.004	**0.0130**	4	0	4	6
Wang et al. (ESRD)	15	0.018	**0.0446**	7	0	7	10
Wang et al. (ESRD)	16	0.032	**0.0640**	4	0	4	6
Wang et al. (ESRD)	17	0.024	**0.0567**	9	0	9	15
Wang et al. (ESRD)	18	0.11	0.1546	4	0	4	7
Wang et al. (ESRD)	19	0.002	**0.0087**	7	0	7	10

Wang et al. (ESRD)	20	0.188	0.2328	14	0	14	22
Wang et al. (ESRD)	21	0.058	0.1005	9	0	9	14
Wang et al. (ESRD)	22	0.076	0.1156	3	0	3	5
Wang et al. (ESRD)	23	0.216	0.2553	3	0	3	5
Wang et al. (ESRD)	24	0.04	**0.0717**	3	0	3	4
Wang et al. (ESRD)	25	0.264	0.2746	5	0	5	7
Wang et al. (ESRD)	26	0.002	**0.0087**	5	0	5	8
Wang et al. (ESRD)	27	0.004	**0.0130**	6	0	6	8
Wang et al. (ESRD)	28	0.07	0.1135	7	0	7	11
Wang et al. (ESRD)	29	0.002	**0.0087**	7	0	7	9
Wang et al. (ESRD)	30	0.002	**0.0087**	7	0	7	11
Wang et al. (ESRD)	31	0.212	0.2553	3	0	3	4
Wang et al. (ESRD)	32	0.142	0.1846	9	0	9	13
Wang et al. (ESRD)	33	0.23	0.2658	3	0	3	5
Wang et al. (ESRD)	34	0.006	**0.0173**	7	0	7	11
Wang et al. (ESRD)	35	0.252	0.2674	16	0	16	23
Wang et al. (ESRD)	36	0.016	**0.0416**	3	0	3	5
Wang et al. (ESRD)	37	0.002	**0.0087**	6	0	6	9
Wang et al. (ESRD)	38	0.002	**0.0087**	4	0	4	7
Wang et al. (ESRD)	39	0.078	0.1156	9	0	9	14
Wang et al. (ESRD)	40	0.026	**0.0588**	3	0	3	4
Wang et al. (ESRD)	41	0.038	**0.0706**	3	0	3	5
Wang et al. (ESRD)	42	0.006	**0.0173**	5	0	5	9
Wang et al. (ESRD)	43	0.002	**0.0087**	6	0	6	8
Wang et al. (ESRD)	44	0.25	0.2674	3	0	3	5
Wang et al. (ESRD)	45	0.03	**0.0640**	3	0	3	5
Wang et al. (ESRD)	46	0.032	**0.0640**	3	0	3	6
Wang et al. (ESRD)	47	0.25	0.2674	4	0	4	6
Wang et al. (ESRD)	48	0.072	0.1135	3	0	3	5
Wang et al. (ESRD)	49	0.002	**0.0087**	7	0	7	11
Wang et al. (ESRD)	50	0.064	0.1074	3	0	3	5
Wang et al. (ESRD)	51	0.002	**0.0087**	5	0	5	8
Wang et al. (ESRD)	52	0.08	0.1156	3	0	3	6

Supplementary Table S6: Detailed modules per dataset

Dataset	Module ID	Node name	Node type	DA p-value	Is an 'anchor' node (p < τ)	Node description
Franzosa et al. (CD)	1	C00062	Metabolite	0.0070891	TRUE	L-Arginine
		C01035	Metabolite	0.0360768	TRUE	4-Guanidinobutanoate
		C00122	Metabolite	NA	FALSE	Fumarate
		EC1.3.1.6	EC	0.0126986	TRUE	fumarate reductase (NADH)
		EC2.1.4.1	EC	0.4632806	FALSE	glycine amidinotransferase
		EC3.5.3.7	EC	0.0156365	TRUE	guanidinobutyrase
		EC4.3.2.1	EC	0.0758779	FALSE	argininosuccinate lyase
		EC6.1.1.19	EC	0.0181257	TRUE	arginine--tRNA ligase
Franzosa et al. (CD)	2	C00064	Metabolite	0.0070931	TRUE	L-Glutamine
		C00534	Metabolite	0.0000044	TRUE	Pyridoxamine
		C00018	Metabolite	NA	FALSE	Pyridoxal phosphate
		C00117	Metabolite	NA	FALSE	D-Ribose 5-phosphate
		C00119	Metabolite	NA	FALSE	5-Phospho-alpha-D-ribose 1-diphosphate
		EC2.4.2.17	EC	0.0000068	TRUE	ATP phosphoribosyltransferase
		EC2.7.1.15	EC	0.0009125	TRUE	ribokinase
		EC2.7.1.35	EC	0.0139983	TRUE	pyridoxal kinase
		EC2.7.6.1	EC	0.0022334	TRUE	ribose-phosphate diphosphokinase
		EC4.3.3.6	EC	0.0121749	TRUE	pyridoxal 5'-phosphate synthase (glutamine hydrolyzing)
		EC5.3.1.6	EC	0.007103	TRUE	ribose-5-phosphate isomerase
		EC6.1.1.18	EC	0.0004638	TRUE	glutamine--tRNA ligase
		EC6.3.1.2	EC	0.0143194	TRUE	glutamine synthetase
		EC6.3.5.5	EC	0.0001033	TRUE	carbamoyl-phosphate synthase (glutamine-hydrolyzing)
Franzosa et al. (CD)	3	C00073	Metabolite	0.1207388	TRUE	L-Methionine
		C00491	Metabolite	0.0000001	TRUE	L-Cystine
		C02989	Metabolite	0.0524836	TRUE	L-Methionine S-oxide
		C00155	Metabolite	NA	FALSE	L-Homocysteine
		EC1.8.4.14	EC	0.2782024	FALSE	L-methionine (R)-S-oxide reductase
		EC2.5.1.49	EC	0.0112205	TRUE	O-acetylhomoserine aminocarboxypropyltransferase
		EC4.4.1.13	EC	0.0459387	FALSE	cysteine-S-conjugate beta-lyase
Franzosa et al. (CD)	5	C00123	Metabolite	0.0083732	TRUE	L-Leucine
		C00141	Metabolite	0.1351861	TRUE	3-Methyl-2-oxobutanoic acid
		C00183	Metabolite	0.0080825	TRUE	L-Valine
		C00407	Metabolite	0.171816	TRUE	L-Isoleucine
		C00109	Metabolite	NA	FALSE	2-Oxobutanoate
		EC1.1.1.85	EC	0.0011663	TRUE	3-isopropylmalate dehydrogenase
		EC2.6.1.42	EC	0.783	FALSE	branched-chain-amino-acid transaminase
Franzosa et al. (CD)	7	C00147	Metabolite	0.0001013	TRUE	Adenine
		C00242	Metabolite	0.0052261	TRUE	Guanine
		C00253	Metabolite	0.0000555	TRUE	Nicotinate
		C00299	Metabolite	0.1938766	TRUE	Uridine
		C00380	Metabolite	0.0260621	TRUE	Cytosine
		C00385	Metabolite	0.1310927	TRUE	Xanthine
		C00559	Metabolite	0.0053919	TRUE	Deoxyadenosine
		C05512	Metabolite	0.0997919	TRUE	Deoxyinosine
		C00130	Metabolite	NA	FALSE	IMP
		C00144	Metabolite	NA	FALSE	GMP
		C04677	Metabolite	NA	FALSE	1-(5'-Phosphoribosyl)-5-amino-4-imidazolecarboxamide
		C00330	Metabolite	NA	FALSE	Deoxyguanosine
		EC1.7.1.7	EC	0.0233303	TRUE	GMP reductase
		EC2.1.2.3	EC	0.0037219	TRUE	phosphoribosylaminoimidazolecarboxamide formyltransferase
		EC2.4.2.1	EC	0.8752706	FALSE	purine-nucleoside phosphorylase
		EC2.4.2.2	EC	0.0088412	TRUE	pyrimidine-nucleoside phosphorylase
		EC2.4.2.7	EC	0.0129047	TRUE	adenine phosphoribosyltransferase
		EC2.4.2.8	EC	0.0006729	TRUE	hypoxanthine phosphoribosyltransferase
		EC2.7.1.113	EC	0.0036545	TRUE	deoxyguanosine kinase
		EC2.7.1.76	EC	0.0061342	TRUE	deoxyadenosine kinase
		EC3.1.5.1	EC	0.0031159	TRUE	dGTPase
		EC3.2.2.1	EC	0.0208245	TRUE	purine nucleosidase
		EC3.2.2.3	EC	0.0095846	TRUE	uridine nucleosidase
		EC3.5.4.10	EC	0.0004162	TRUE	IMP cyclohydrolase
		EC3.5.4.4	EC	0.0036506	TRUE	adenosine deaminase
		EC6.3.5.2	EC	0.0054071	TRUE	GMP synthase (glutamine-hydrolyzing)
Franzosa et al. (CD)	9	C00163	Metabolite	0.1354209	TRUE	Propanoate
		C00186	Metabolite	0.0001325	TRUE	(S)-Lactate
		C05984	Metabolite	0.0019751	TRUE	2-Hydroxybutanoic acid
		EC1.1.1.27	EC	0.1285315	FALSE	L-lactate dehydrogenase
		EC2.8.3.1	EC	0.1141363	FALSE	propionate CoA-transferase
Franzosa et al. (CD)	11	C00219	Metabolite	0.0000921	TRUE	Arachidonate
Franzosa et al. (CD)	11	C01595	Metabolite	0.1006297	TRUE	Linoleate
		C06427	Metabolite	0.0085016	TRUE	(9Z,12Z,15Z)-Octadecatrienoic acid
		C04317	Metabolite	NA	FALSE	1-Organyl-2-lyso-sn-glycero-3-phosphocholine
		EC3.1.1.4	EC	0.5028989	FALSE	phospholipase A2
		EC3.1.1.47	EC	0.0200866	TRUE	1-alkyl-2-acetylglycerophosphocholine esterase
Franzosa et al. (CD)	12	C00245	Metabolite	0.0004625	TRUE	Taurine
		C00695	Metabolite	0.0000955	TRUE	Cholic acid
		C01921	Metabolite	0.0109106	TRUE	Glycocholate
		C02528	Metabolite	0.000013	TRUE	Chenodeoxycholate
		C05465	Metabolite	0.10341	TRUE	Taurochenodeoxycholate
		EC3.5.1.24	EC	0.1483136	FALSE	choloylglycine hydrolase
Franzosa et al. (CD)	13	C00255	Metabolite	0.0223581	TRUE	Riboflavin
		C00378	Metabolite	0.1602965	TRUE	Thiamine
		C00870	Metabolite	0.0745124	TRUE	4-Nitrophenol
		C00016	Metabolite	NA	FALSE	FAD
		EC1.5.1.36	EC	0.003229	TRUE	flavin reductase (NADH)

		EC2.7.7.2	EC	0.0194212	TRUE	FAD synthase
		EC3.1.3.1	EC	0.0021371	TRUE	alkaline phosphatase
		EC3.1.3.2	EC	0.073548	FALSE	acid phosphatase
Franzosa et al. (CD)	15	C00300	Metabolite	0.0133647	TRUE	Creatine
		C00719	Metabolite	0.0956604	TRUE	Betaine
		C00213	Metabolite	NA	FALSE	Sarcosine
		EC2.1.1.157	EC	0.0063813	TRUE	sarcosine/dimethylglycine N-methyltransferase
		EC3.5.3.3	EC	NA	FALSE	creatinase
Franzosa et al. (CD)	17	C05607	Metabolite	0.0074119	TRUE	Phenyllactate
		C00166	Metabolite	NA	FALSE	Phenylpyruvate
		C01179	Metabolite	NA	FALSE	3-(4-Hydroxyphenyl)pyruvate
		C00133	Metabolite	NA	FALSE	D-Alanine
		EC1.13.11.27	EC	0.0101889	TRUE	4-hydroxyphenylpyruvate dioxygenase
		EC1.3.1.12	EC	0.0121343	TRUE	prephenate dehydrogenase
		EC2.6.1.1	EC	0.0108993	TRUE	aspartate transaminase
		EC2.6.1.21	EC	0.0141202	TRUE	D-amino-acid transaminase
		EC4.2.1.51	EC	0.0058454	TRUE	prephenate dehydratase
		EC6.1.1.13	EC	0.0052979	TRUE	D-alanine--poly(phosphoribitol) ligase
		EC1.1.1.110	EC	NA	FALSE	aromatic 2-oxoacid reductase
Franzosa et al. (CD)	21	C00063	Metabolite	NA	FALSE	CTP
		C00143	Metabolite	NA	FALSE	5,10-Methylenetetrahydrofolate
		C00415	Metabolite	NA	FALSE	Dihydrofolate
		C00364	Metabolite	NA	FALSE	dTMP
		C00103	Metabolite	NA	FALSE	D-Glucose 1-phosphate
		C00857	Metabolite	NA	FALSE	Deamino-NAD+
		EC1.17.4.2	EC	0.0107003	TRUE	ribonucleoside-triphosphate reductase (thioredoxin)
		EC1.5.1.3	EC	0.0000004	TRUE	dihydrofolate reductase
		EC1.5.1.5	EC	0.0176342	TRUE	methylenetetrahydrofolate dehydrogenase (NADP(+))
		EC2.1.1.148	EC	0.0007374	TRUE	thymidylate synthase (FAD)
		EC2.1.1.45	EC	0.0000025	TRUE	thymidylate synthase
		EC2.4.1.20	EC	0.0000072	TRUE	cellobiose phosphorylase
		EC2.4.1.7	EC	0.0069104	TRUE	sucrose phosphorylase
		EC2.7.7.18	EC	0.0001487	TRUE	nicotinate-nucleotide adenylyltransferase
		EC2.7.7.40	EC	0.0180712	TRUE	D-ribitol-5-phosphate cytidylyltransferase
		EC2.7.7.9	EC	0.0071367	TRUE	UTP--glucose-1-phosphate uridylyltransferase
		EC3.6.1.9	EC	0.0449158	FALSE	nucleotide diphosphatase
		EC6.3.5.1	EC	0.0158834	TRUE	NAD(+) synthase (glutamine-hydrolyzing)
Franzosa et al. (CD)	22	C00085	Metabolite	NA	FALSE	D-Fructose 6-phosphate
		C00092	Metabolite	NA	FALSE	D-Glucose 6-phosphate
		C00275	Metabolite	NA	FALSE	D-Mannose 6-phosphate
		EC2.2.1.2	EC	0.0194758	TRUE	transaldolase
		EC2.7.1.191	EC	0.0032159	TRUE	protein-N(pi)-phosphohistidine--D-mannose phosphotransferase
		EC3.2.1.86	EC	0.0069758	TRUE	6-phospho-beta-glucosidase
		EC3.5.99.6	EC	0.0130802	TRUE	glucosamine-6-phosphate deaminase
		EC5.3.1.8	EC	0.008899	TRUE	mannose-6-phosphate isomerase
		EC5.4.2.6	EC	0.0055886	TRUE	beta-phosphoglucomutase
		EC2.7.1.1	EC	NA	FALSE	hexokinase
Franzosa et al. (CD)	26	C00357	Metabolite	NA	FALSE	N-Acetyl-D-glucosamine 6-phosphate
		EC3.5.1.25	EC	0.0011891	TRUE	N-acetylglucosamine-6-phosphate deacetylase
		EC4.2.1.126	EC	0.0005033	TRUE	N-acetylmuramic acid 6-phosphate etherase
		EC5.1.3.9	EC	0.0005362	TRUE	N-acylglucosamine-6-phosphate 2-epimerase
Franzosa et al. (UC)	1	C00047	Metabolite	0.0487764	TRUE	L-Lysine
		C00740	Metabolite	NA	FALSE	D-Serine
		C00793	Metabolite	NA	FALSE	D-Cysteine
		EC4.1.1.18	EC	0.0200206	TRUE	lysine decarboxylase
		EC4.3.1.18	EC	0.0023189	TRUE	D-serine ammonia-lyase
		EC4.4.1.15	EC	0.0010792	TRUE	D-cysteine desulfhydrase
		EC6.1.1.6	EC	0.0411433	TRUE	lysine--tRNA ligase
		EC5.1.1.10	EC	NA	FALSE	amino-acid racemase
Franzosa et al. (UC)	2	C00073	Metabolite	0.0456204	TRUE	L-Methionine
		C02989	Metabolite	0.039172	TRUE	L-Methionine S-oxide
		C01179	Metabolite	NA	FALSE	3-(4-Hydroxyphenyl)pyruvate
		C00826	Metabolite	NA	FALSE	L-Arogenate
		C05703	Metabolite	NA	FALSE	Methaneselenol
		C00155	Metabolite	NA	FALSE	L-Homocysteine
		EC1.13.11.27	EC	0.0056707	TRUE	4-hydroxyphenylpyruvate dioxygenase
		EC1.3.1.43	EC	0.013479	TRUE	arogenate dehydrogenase
		EC1.8.1.9	EC	0.0873579	TRUE	thioredoxin-disulfide reductase
		EC1.8.4.14	EC	0.015776	TRUE	L-methionine (R)-S-oxide reductase
		EC2.1.1.14	EC	0.0819209	TRUE	5-methyltetrahydropteroyltriglutamate--homocysteine S-methyltransferase
		EC2.5.1.49	EC	0.0000364	TRUE	O-acetylhomoserine aminocarboxypropyltransferase
		EC2.5.1.6	EC	0.0320355	TRUE	methionine adenosyltransferase
		EC2.6.1.1	EC	0.0806571	TRUE	aspartate transaminase
		EC2.6.1.57	EC	0.0082221	TRUE	aromatic-amino-acid transaminase
		EC2.6.1.88	EC	0.0038696	TRUE	methionine transaminase
		EC2.6.1.9	EC	0.0537561	TRUE	histidinol-phosphate transaminase
		EC4.4.1.11	EC	0.0232202	TRUE	methionine gamma-lyase
		EC4.4.1.13	EC	0.0189139	TRUE	cysteine-S-conjugate beta-lyase
		EC4.4.1.21	EC	0.0767176	TRUE	S-ribosylhomocysteine lyase
		EC6.1.1.10	EC	0.0068558	TRUE	methionine--tRNA ligase
Franzosa et al. (UC)	5	C00163	Metabolite	0.0018882	TRUE	Propanoate
		C00186	Metabolite	0.0299332	TRUE	(S)-Lactate
		EC1.1.1.27	EC	0.072757	TRUE	L-lactate dehydrogenase
		EC1.1.2.3	EC	0.02873	TRUE	L-lactate dehydrogenase (cytochrome)
		EC1.2.1.22	EC	0.015001	TRUE	lactaldehyde dehydrogenase
		EC2.7.2.1	EC	0.0185007	TRUE	acetate kinase
		EC2.7.2.15	EC	0.0110678	TRUE	propionate kinase
		EC2.8.3.1	EC	0.0005889	TRUE	propionate CoA-transferase
		EC6.2.1.1	EC	0.0220136	TRUE	acetate--CoA ligase
		EC6.2.1.13	EC	0.0046118	TRUE	acetate--CoA ligase (ADP-forming)

		EC6.2.1.17	EC	0.0167088	TRUE	propionate--CoA ligase
Franzosa et al. (UC)	6	C00106	Metabolite	0.1467285	FALSE	Uracil
		C00178	Metabolite	0.0228594	TRUE	Thymine
		C00262	Metabolite	0.0372418	TRUE	Hypoxanthine
		C00385	Metabolite	0.048851	TRUE	Xanthine
		C00387	Metabolite	0.0819116	FALSE	Guanosine
		C00559	Metabolite	0.0036766	TRUE	Deoxyadenosine
		C05512	Metabolite	0.0083269	TRUE	Deoxyinosine
		C00130	Metabolite	NA	FALSE	IMP
		C00620	Metabolite	NA	FALSE	alpha-D-Ribose 1-phosphate
		C00330	Metabolite	NA	FALSE	Deoxyguanosine
		C00475	Metabolite	NA	FALSE	Cytidine
		C11736	Metabolite	NA	FALSE	5-Fluorodeoxyuridine
		EC1.14.99.46	EC	0.0137342	TRUE	pyrimidine monooxygenase
		EC1.17.1.4	EC	0.0444586	TRUE	xanthine dehydrogenase
		EC1.7.1.7	EC	0.0243602	TRUE	GMP reductase
		EC2.4.2.15	EC	0.0246241	TRUE	guanosine phosphorylase
		EC2.4.2.2	EC	0.0030429	TRUE	pyrimidine-nucleoside phosphorylase
		EC2.4.2.4	EC	0.0053371	TRUE	thymidine phosphorylase
		EC2.7.1.21	EC	0.0412729	TRUE	thymidine kinase
		EC2.7.1.48	EC	0.0255492	TRUE	uridine/cytidine kinase
		EC2.7.1.73	EC	0.0070133	TRUE	inosine kinase
		EC3.1.3.6	EC	0.0154348	TRUE	3'-nucleotidase
		EC3.1.3.89	EC	0.0054584	TRUE	5'-deoxynucleotidase
		EC3.1.5.1	EC	0.0131713	TRUE	dGTPase
		EC3.2.2.8	EC	0.0033516	TRUE	ribosylpyrimidine nucleosidase
		EC3.5.4.1	EC	0.0020373	TRUE	cytosine deaminase
		EC3.5.4.10	EC	0.0757455	TRUE	IMP cyclohydrolase
		EC3.5.4.2	EC	0.0102749	TRUE	adenine deaminase
		EC3.6.1.66	EC	0.0074313	TRUE	XTP/dITP diphosphatase
		EC4.2.1.70	EC	0.010587	TRUE	pseudouridylate synthase
Franzosa et al. (UC)	8	C00219	Metabolite	0.0009147	TRUE	Arachidonate
		C01595	Metabolite	0.0131372	TRUE	Linoleate
		C00350	Metabolite	NA	FALSE	Phosphatidylethanolamine
		EC2.3.1.40	EC	0.0132239	TRUE	acyl-[acyl-carrier-protein]--phospholipid O-acyltransferase
		EC3.1.1.32	EC	0.0042317	TRUE	phospholipase A1
		EC3.1.1.4	EC	0.0112875	TRUE	phospholipase A2
		EC4.1.1.65	EC	0.0027152	TRUE	phosphatidylserine decarboxylase
Franzosa et al. (UC)	14	C06423	Metabolite	0.005357	TRUE	Octanoic acid
		C15972	Metabolite	NA	FALSE	Enzyme N6-(lipoyl)lysine
		C02051	Metabolite	NA	FALSE	Lipoylprotein
		C15973	Metabolite	NA	FALSE	Enzyme N6-(dihydrolipoyl)lysine
		C22160	Metabolite	NA	FALSE	[Lipoyl-carrier protein E2]-N6-octanoyl-L-lysine
		EC1.2.4.1	EC	0.0020826	TRUE	pyruvate dehydrogenase (acetyl-transferring)
		EC1.2.4.2	EC	0.0045247	TRUE	oxoglutarate dehydrogenase (succinyl-transferring)
		EC1.4.4.2	EC	0.0009302	TRUE	glycine dehydrogenase (aminomethyl-transferring)
		EC1.8.1.4	EC	0.000527	TRUE	dihydrolipoyl dehydrogenase
		EC2.3.1.12	EC	0.0037579	TRUE	dihydrolipoyllysine-residue acetyltransferase
		EC2.3.1.181	EC	0.0542052	TRUE	lipoyl(octanoyl) transferase
		EC2.3.1.61	EC	0.0063099	TRUE	dihydrolipoyllysine-residue succinyltransferase
		EC2.8.1.8	EC	0.0068629	TRUE	lipoyl synthase
		EC6.3.1.20	EC	0.0018009	TRUE	lipoate--protein ligase
Franzosa et al. (UC)	18	C00085	Metabolite	NA	FALSE	D-Fructose 6-phosphate
		C01172	Metabolite	NA	FALSE	beta-D-Glucose 6-phosphate
		C00275	Metabolite	NA	FALSE	D-Mannose 6-phosphate
		EC1.1.1.140	EC	0.0085311	TRUE	sorbitol-6-phosphate 2-dehydrogenase
		EC1.1.1.17	EC	0.0469013	TRUE	mannitol-1-phosphate 5-dehydrogenase
		EC2.2.1.1	EC	0.026545	TRUE	transketolase
		EC2.2.1.2	EC	0.0462893	TRUE	transaldolase
		EC2.7.1.191	EC	0.0029294	TRUE	protein-N(pi)-phosphohistidine--D-mannose phosphotransferase
		EC2.7.1.90	EC	0.0691716	TRUE	diphosphate--fructose-6-phosphate 1-phosphotransferase
		EC3.2.1.86	EC	0.0068317	TRUE	6-phospho-beta-glucosidase
		EC5.1.3.15	EC	0.012739	TRUE	glucose-6-phosphate 1-epimerase
		EC5.3.1.8	EC	0.0124942	TRUE	mannose-6-phosphate isomerase
		EC2.7.1.1	EC	NA	FALSE	hexokinase
Franzosa et al. (UC)	23	C00168	Metabolite	NA	FALSE	Hydroxypyruvate
		C00160	Metabolite	NA	FALSE	Glycolate
		C01146	Metabolite	NA	FALSE	2-Hydroxy-3-oxopropanoate
		EC1.1.1.29	EC	0.0447201	TRUE	glycerate dehydrogenase
		EC1.1.1.60	EC	0.023852	TRUE	2-hydroxy-3-oxopropionate reductase
		EC1.1.1.79	EC	0.0168131	TRUE	glyoxylate reductase (NADP(+))
		EC1.1.1.81	EC	0.0231383	TRUE	hydroxypyruvate reductase
		EC3.1.3.18	EC	0.0766582	TRUE	phosphoglycolate phosphatase
		EC3.8.1.2	EC	0.0857366	TRUE	(S)-2-haloacid dehalogenase
		EC3.8.1.3	EC	0.0023936	TRUE	haloacetate dehalogenase
		EC4.1.1.47	EC	0.0088281	TRUE	tartronate-semialdehyde synthase
		EC5.3.1.22	EC	0.0068332	TRUE	hydroxypyruvate isomerase
Franzosa et al. (UC)	24	C00099	Metabolite	0.1843589	FALSE	beta-Alanine
		C00222	Metabolite	NA	FALSE	3-Oxopropanoate
		C00080	Metabolite	NA	FALSE	H+
		C12455	Metabolite	NA	FALSE	5-Aminopentanal
		C00334	Metabolite	NA	FALSE	4-Aminobutanoate
		EC1.1.1.298	EC	0.0304935	TRUE	3-hydroxypropionate dehydrogenase (NADP(+))
		EC1.17.1.9	EC	0.0047978	TRUE	formate dehydrogenase
		EC1.2.1.19	EC	0.0016446	TRUE	aminobutyraldehyde dehydrogenase
		EC1.2.1.27	EC	0.0810232	TRUE	methylmalonate-semialdehyde dehydrogenase (CoA acylating)
		EC2.6.1.19	EC	0.0052149	TRUE	4-aminobutyrate--2-oxoglutarate transaminase
		EC2.6.1.82	EC	0.0061111	TRUE	putrescine--2-oxoglutarate transaminase
		EC3.5.3.7	EC	0.0075329	TRUE	guanidinobutyrase
		EC4.1.1.11	EC	0.0387885	TRUE	aspartate 1-decarboxylase

		EC4.1.1.15	EC	0.0054334	TRUE	glutamate decarboxylase
		EC4.1.2.29	EC	0.03134	TRUE	5-dehydro-2-deoxyphosphogluconate aldolase
Franzosa et al. (UC)	41	C00136	Metabolite	NA	FALSE	Butanoyl-CoA
		C05271	Metabolite	NA	FALSE	trans-Hex-2-enoyl-CoA
		C00877	Metabolite	NA	FALSE	Crotonoyl-CoA
		C00894	Metabolite	NA	FALSE	Propenoyl-CoA
		EC1.2.1.10	EC	0.0133554	TRUE	acetaldehyde dehydrogenase (acetylating)
		EC1.3.1.84	EC	0.0857759	TRUE	acrylyl-CoA reductase (NADPH)
		EC1.3.8.1	EC	0.0598369	TRUE	short-chain acyl-CoA dehydrogenase
		EC1.3.8.7	EC	0.0041613	TRUE	medium-chain acyl-CoA dehydrogenase
		EC1.3.8.8	EC	0.0041613	TRUE	long-chain-acyl-CoA dehydrogenase
		EC2.8.3.12	EC	0.0932126	TRUE	glutaconate CoA-transferase
		EC2.8.3.8	EC	0.0313603	TRUE	acetate CoA-transferase
		EC2.8.3.9	EC	0.0036001	TRUE	butyrate--acetoacetate CoA-transferase
		EC7.2.4.5	EC	0.0044418	TRUE	glutaconyl-CoA decarboxylase
Franzosa et al. (UC)	45	C04171	Metabolite	NA	FALSE	(2S,3S)-2,3-Dihydro-2,3-dihydroxybenzoate
		C00885	Metabolite	NA	FALSE	Isochorismate
		C16519	Metabolite	NA	FALSE	2-Succinyl-5-enolpyruvyl-6-hydroxy-3-cyclohexene-1-carboxylate
		EC1.3.1.28	EC	0.014745	TRUE	2,3-dihydro-2,3-dihydroxybenzoate dehydrogenase
		EC2.2.1.9	EC	0.0441052	TRUE	2-succinyl-5-enolpyruvyl-6-hydroxy-3-cyclohexene-1-carboxylic-acid
		EC3.3.2.1	EC	0.0047293	TRUE	isochorismatase
		EC4.2.99.20	EC	0.0154522	TRUE	2-succinyl-6-hydroxy-2,4-cyclohexadiene-1-carboxylate synthase
Franzosa et al. (UC)	46	C05761	Metabolite	NA	FALSE	Tetradecanoyl-[acp]
		C06025	Metabolite	NA	FALSE	KDO2-lipid IVA
		C00229	Metabolite	NA	FALSE	Acyl-carrier protein
		C00010	Metabolite	NA	FALSE	CoA
		EC1.3.1.9	EC	0.0921544	TRUE	enoyl-[acyl-carrier-protein] reductase (NADH)
		EC2.3.1.241	EC	0.0508634	TRUE	Kdo2-lipid IVA acyltransferase
		EC2.3.1.242	EC	0.0057361	TRUE	Kdo2-lipid IVA palmitoleoyltransferase
		EC2.3.1.243	EC	0.0245015	TRUE	acyl-Kdo2-lipid IVA acyltransferase
		EC2.7.1.24	EC	0.0553571	TRUE	dephospho-CoA kinase
		EC2.7.8.7	EC	0.0119738	TRUE	holo-[acyl-carrier-protein] synthase
		EC3.1.4.14	EC	0.0057125	TRUE	[acyl-carrier-protein] phosphodiesterase
		EC6.2.1.20	EC	0.0320389	TRUE	long-chain-fatty-acid--[acyl-carrier-protein] ligase
Franzosa et al. (UC)	48	C00143	Metabolite	NA	FALSE	5,10-Methylenetetrahydrofolate
		C00445	Metabolite	NA	FALSE	5,10-Methenyltetrahydrofolate
		C00365	Metabolite	NA	FALSE	dUMP
		EC1.5.1.5	EC	0.0260344	TRUE	methylenetetrahydrofolate dehydrogenase (NADP(+))
		EC2.1.1.148	EC	0.0103823	TRUE	thymidylate synthase (FAD)
		EC2.1.1.45	EC	0.0478156	TRUE	thymidylate synthase
		EC2.1.2.10	EC	0.0023197	TRUE	aminomethyltransferase
		EC3.5.4.12	EC	0.0913597	TRUE	dCMP deaminase
		EC3.5.4.9	EC	0.0825722	TRUE	methenyltetrahydrofolate cyclohydrolase
		EC6.3.3.2	EC	0.0021834	TRUE	5-formyltetrahydrofolate cyclo-ligase
Franzosa et al. (UC)	61	C00288	Metabolite	NA	FALSE	HCO3-
		C01563	Metabolite	NA	FALSE	Carbamate
		EC2.7.2.2	EC	0.0221094	TRUE	carbamate kinase
		EC3.5.1.110	EC	0.0084051	TRUE	ureidoacrylate amidohydrolase
		EC4.2.1.1	EC	0.0310636	TRUE	carbonic anhydrase
		EC6.4.1.2	EC	0.0137271	TRUE	acetyl-CoA carboxylase
Jeffery et al. (IBS)	2	C00047	Metabolite	0.00455	TRUE	L-Lysine
		C00062	Metabolite	0.00239	TRUE	L-Arginine
		C00064	Metabolite	0.022	TRUE	L-Glutamine
		C00065	Metabolite	0.0598	TRUE	L-Serine
		C00078	Metabolite	0.0197	TRUE	L-Tryptophan
		C00680	Metabolite	NA	FALSE	meso-2,6-Diaminoheptanedioate
		EC1.4.1.13	EC	0.0043725	TRUE	glutamate synthase (NADPH)
		EC1.4.1.16	EC	0.0315269	TRUE	diaminopimelate dehydrogenase
		EC2.1.2.1	EC	0.1012129	TRUE	glycine hydroxymethyltransferase
		EC2.4.2.14	EC	0.0091258	TRUE	amidophosphoribosyltransferase
		EC2.6.1.16	EC	0.0550353	TRUE	glutamine--fructose-6-phosphate transaminase (isomerizing)
		EC3.1.3.3	EC	0.0246651	TRUE	phosphoserine phosphatase
		EC3.5.3.1	EC	0.0177749	TRUE	arginase
		EC4.1.1.19	EC	0.1049725	TRUE	arginine decarboxylase
		EC4.1.1.20	EC	0.0008834	TRUE	diaminopimelate decarboxylase
		EC4.1.99.1	EC	0.0679459	TRUE	tryptophanase
		EC4.2.1.20	EC	0.0019985	TRUE	tryptophan synthase
		EC4.3.1.17	EC	0.0407892	TRUE	L-serine ammonia-lyase
		EC4.3.1.19	EC	0.0083417	TRUE	threonine ammonia-lyase
		EC4.3.2.1	EC	0.0394089	TRUE	argininosuccinate lyase
		EC5.1.1.7	EC	0.0007209	TRUE	diaminopimelate epimerase
		EC5.4.3.2	EC	0.0070809	TRUE	lysine 2,3-aminomutase
		EC6.3.1.2	EC	0.000353	TRUE	glutamine synthetase
		EC6.3.5.5	EC	0.004354	TRUE	carbamoyl-phosphate synthase (glutamine-hydrolyzing)
		EC5.1.1.10	EC	NA	FALSE	amino-acid racemase
Jeffery et al. (IBS)	4	C00147	Metabolite	0.00362	TRUE	Adenine
		C00153	Metabolite	0.0436	TRUE	Nicotinamide
		C00212	Metabolite	0	TRUE	Adenosine
		C00385	Metabolite	0.992	FALSE	Xanthine
		C00003	Metabolite	NA	FALSE	NAD+
		C00455	Metabolite	NA	FALSE	Nicotinamide D-ribonucleotide
		EC1.17.1.4	EC	0.1086373	TRUE	xanthine dehydrogenase
		EC2.4.2.1	EC	0.1863915	FALSE	purine-nucleoside phosphorylase
		EC2.7.1.22	EC	0.0057711	TRUE	ribosylnicotinamide kinase
		EC2.7.7.1	EC	0.1142293	TRUE	nicotinamide-nucleotide adenylyltransferase
		EC2.7.7.18	EC	0.0268878	TRUE	nicotinate-nucleotide adenylyltransferase
		EC3.2.2.1	EC	0.0009868	TRUE	purine nucleosidase
		EC3.2.2.4	EC	0.04242	TRUE	AMP nucleosidase
		EC3.5.1.19	EC	0.024392	TRUE	nicotinamidase
		EC3.5.4.2	EC	0.0160879	TRUE	adenine deaminase

		EC3.5.4.3	EC	0.0003737	TRUE	guanine deaminase
		EC3.6.1.22	EC	0.0080601	TRUE	NAD(+) diphosphatase
		EC3.2.2.5	EC	NA	FALSE	NAD(+) glycohydrolase
Jeffery et al. (IBS)	16	C01144	Metabolite	NA	FALSE	(S)-3-Hydroxybutanoyl-CoA
		C00332	Metabolite	NA	FALSE	Acetoacetyl-CoA
		C00136	Metabolite	NA	FALSE	Butanoyl-CoA
		C00246	Metabolite	NA	FALSE	Butanoic acid
		EC1.1.1.157	EC	0.0603314	TRUE	3-hydroxybutyryl-CoA dehydrogenase
		EC1.1.1.36	EC	0.0707496	TRUE	acetoacetyl-CoA reductase
		EC1.3.8.1	EC	0.0639693	TRUE	short-chain acyl-CoA dehydrogenase
		EC2.7.2.7	EC	0.0583767	TRUE	butyrate kinase
		EC2.8.3.8	EC	0.0093017	TRUE	acetate CoA-transferase
		EC2.8.3.9	EC	0.0100055	TRUE	butyrate--acetoacetate CoA-transferase
		EC5.1.2.3	EC	0.1233479	TRUE	3-hydroxybutyryl-CoA epimerase
		EC6.2.1.16	EC	0.0463745	TRUE	acetoacetate--CoA ligase
Jeffery et al. (IBS)	33	C00106	Metabolite	0.411	FALSE	Uracil
		C00299	Metabolite	0.736	FALSE	Uridine
		C21028	Metabolite	NA	FALSE	(R)-5,6-Dihydrothymine
		C00365	Metabolite	NA	FALSE	dUMP
		C00526	Metabolite	NA	FALSE	Deoxyuridine
		C00105	Metabolite	NA	FALSE	UMP
		EC1.3.1.1	EC	0.0459956	TRUE	dihydrouracil dehydrogenase (NAD(+))
		EC2.1.1.148	EC	0.1097752	TRUE	thymidylate synthase (FAD)
		EC2.4.2.3	EC	0.0075441	TRUE	uridine phosphorylase
		EC2.4.2.9	EC	0.1003691	TRUE	uracil phosphoribosyltransferase
		EC2.7.1.21	EC	0.0771583	TRUE	thymidine kinase
		EC3.2.2.8	EC	0.104194	TRUE	ribosylpyrimidine nucleosidase
		EC3.5.2.2	EC	0.0112293	TRUE	dihydropyrimidinase
		EC3.5.4.12	EC	0.0486858	TRUE	dCMP deaminase
		EC3.5.4.5	EC	0.0234026	TRUE	cytidine deaminase
		EC4.1.1.23	EC	0.0209007	TRUE	orotidine-5'-phosphate decarboxylase
Jeffery et al. (IBS)	38	C00255	Metabolite	0.0716	FALSE	Riboflavin
		C01847	Metabolite	NA	FALSE	Reduced FMN
		C00870	Metabolite	NA	FALSE	4-Nitrophenol
		C00061	Metabolite	NA	FALSE	FMN
		C00378	Metabolite	NA	FALSE	Thiamine
		C01081	Metabolite	NA	FALSE	Thiamin monophosphate
		EC1.5.1.38	EC	0.1278402	TRUE	FMN reductase (NADPH)
		EC1.5.1.39	EC	0.0000592	TRUE	FMN reductase [NAD(P)H]
		EC2.5.1.129	EC	0.0442198	TRUE	flavin prenyltransferase
		EC2.5.1.3	EC	0.0195021	TRUE	thiamine phosphate synthase
		EC2.5.1.9	EC	0.1279267	TRUE	riboflavin synthase
		EC2.7.6.2	EC	0.0029873	TRUE	thiamine diphosphokinase
		EC3.1.3.1	EC	0.0076504	TRUE	alkaline phosphatase
		EC3.1.3.2	EC	0.0204965	TRUE	acid phosphatase
		EC3.1.8.1	EC	0.0035917	TRUE	aryldialkylphosphatase
Jeffery et al. (IBS)	40	C02463	Metabolite	NA	FALSE	Precorrin 2
		C01051	Metabolite	NA	FALSE	Uroporphyrinogen III
		EC2.1.1.107	EC	0.0165905	TRUE	uroporphyrinogen-III C-methyltransferase
		EC2.1.1.130	EC	0.1071368	TRUE	precorrin-2 C(20)-methyltransferase
		EC4.1.1.37	EC	0.027818	TRUE	uroporphyrinogen decarboxylase
Jeffery et al. (IBS)	47	C19848	Metabolite	NA	FALSE	Adenylated molybdopterin
		C18237	Metabolite	NA	FALSE	Molybdoenzyme molybdenum cofactor
		EC2.10.1.1	EC	0.0095764	TRUE	molybdopterin molybdotransferase
		EC2.7.7.75	EC	0.0032074	TRUE	molybdopterin adenylyltransferase
		EC2.7.7.77	EC	0.000755	TRUE	molybdenum cofactor guanylyltransferase
Jeffery et al. (IBS)	48	C11437	Metabolite	NA	FALSE	1-Deoxy-D-xylulose 5-phosphate
		C15809	Metabolite	NA	FALSE	Iminoglycine
		EC2.2.1.7	EC	0.0680482	TRUE	1-deoxy-D-xylulose-5-phosphate synthase
		EC2.8.1.10	EC	0.0507634	TRUE	thiazole synthase
		EC4.1.99.19	EC	0.0003403	TRUE	2-iminoacetate synthase
Jeffery et al. (IBS)	51	C01092	Metabolite	NA	FALSE	8-Amino-7-oxononanoate
		C01037	Metabolite	NA	FALSE	7,8-Diaminononanoate
		C01909	Metabolite	NA	FALSE	Dethiobiotin
		EC2.3.1.47	EC	0.0049038	TRUE	8-amino-7-oxononanoate synthase
		EC2.6.1.62	EC	0.0001355	TRUE	adenosylmethionine--8-amino-7-oxononanoate transaminase
		EC2.8.1.6	EC	0.0040822	TRUE	biotin synthase
		EC6.3.3.3	EC	0.0036338	TRUE	dethiobiotin synthase
Jeffery et al. (IBS)	53	C00029	Metabolite	NA	FALSE	UDP-glucose
		C00369	Metabolite	NA	FALSE	Starch
		C00103	Metabolite	NA	FALSE	D-Glucose 1-phosphate
		C00089	Metabolite	NA	FALSE	Sucrose
		C00718	Metabolite	NA	FALSE	Amylose
		C00052	Metabolite	NA	FALSE	UDP-alpha-D-galactose
		C01935	Metabolite	NA	FALSE	Maltodextrin
		EC2.4.1.1	EC	0.065038	TRUE	glycogen phosphorylase
		EC2.4.1.11	EC	0.0891718	TRUE	glycogen(starch) synthase
		EC2.4.1.18	EC	0.0520558	TRUE	1,4-alpha-glucan branching enzyme
		EC2.4.1.20	EC	0.0561356	TRUE	cellobiose phosphorylase
		EC2.4.1.21	EC	0.0206606	TRUE	starch synthase
		EC2.4.1.4	EC	0.0084425	TRUE	amylosucrase
		EC2.4.1.5	EC	0.0094693	TRUE	dextransucrase
		EC2.4.1.7	EC	0.0537858	TRUE	sucrose phosphorylase
		EC2.4.99.16	EC	0.1190859	TRUE	starch synthase (maltosyl-transferring)
		EC2.7.7.27	EC	0.0990762	TRUE	glucose-1-phosphate adenylyltransferase
		EC2.7.7.9	EC	0.0412613	TRUE	UTP--glucose-1-phosphate uridylyltransferase
		EC3.2.1.1	EC	0.0042664	TRUE	alpha-amylase
		EC3.2.1.3	EC	0.106752	TRUE	glucan 1,4-alpha-glucosidase
		EC3.2.1.54	EC	0.0055335	TRUE	cyclomaltodextrinase
		EC3.2.1.68	EC	0.0819147	TRUE	isoamylase

		EC3.6.1.45	EC	0.1182435	TRUE	UDP-sugar diphosphatase
		EC5.1.3.2	EC	0.0002219	TRUE	UDP-glucose 4-epimerase
		EC5.4.99.9	EC	0.0005754	TRUE	UDP-galactopyranose mutase
Jeffery et al. (IBS)	59	C00129	Metabolite	NA	FALSE	Isopentenyl diphosphate
		EC2.5.1.1	EC	0.0095195	TRUE	dimethylallyltranstransferase
		EC2.5.1.10	EC	0.0014252	TRUE	(2E,6E)-farnesyl diphosphate synthase
		EC2.5.1.29	EC	0.0024533	TRUE	geranylgeranyl diphosphate synthase
		EC5.3.3.2	EC	0.0040147	TRUE	isopentenyl-diphosphate Delta-isomerase
Jeffery et al. (IBS)	60	C06508	Metabolite	NA	FALSE	Adenosyl cobinamide
		C06509	Metabolite	NA	FALSE	Adenosyl cobinamide phosphate
		C04122	Metabolite	NA	FALSE	D-1-Aminopropan-2-ol O-phosphate
		EC2.5.1.17	EC	0.0007645	TRUE	corrinoid adenosyltransferase
		EC2.7.1.156	EC	0.0060653	TRUE	adenosylcobinamide kinase
		EC2.7.7.62	EC	0.0133206	TRUE	adenosylcobinamide-phosphate guanylyltransferase
		EC4.1.1.81	EC	0.0004845	TRUE	threonine-phosphate decarboxylase
		EC6.3.1.10	EC	NA	FALSE	adenosylcobinamide-phosphate synthase
Jeffery et al. (IBS)	68	C00086	Metabolite	NA	FALSE	Urea
		C01010	Metabolite	NA	FALSE	Urea-1-carboxylate
		EC3.5.1.5	EC	0.0012855	TRUE	urease
		EC3.5.1.54	EC	0.0280354	TRUE	allophanate hydrolase
		EC6.3.4.6	EC	0.1367414	TRUE	urea carboxylase
Wang et al. (ESRD)	6	C00188	Metabolite	NA	FALSE	L-Threonine
		C03508	Metabolite	NA	FALSE	L-2-Amino-3-oxobutanoic acid
		EC1.1.1.103	EC	0.0011409	TRUE	L-threonine 3-dehydrogenase
		EC2.3.1.29	EC	0.0011881	TRUE	glycine C-acetyltransferase
		EC4.1.2.48	EC	0.0715074	TRUE	low-specificity L-threonine aldolase
		EC4.2.3.1	EC	0.0017932	TRUE	threonine synthase
Wang et al. (ESRD)	7	C00310	Metabolite	NA	FALSE	D-Xylulose
		C00181	Metabolite	NA	FALSE	D-Xylose
		C00267	Metabolite	NA	FALSE	alpha-D-Glucose
		EC1.1.1.14	EC	0.0812683	TRUE	L-iditol 2-dehydrogenase
		EC1.1.1.179	EC	0.0505813	TRUE	D-xylose 1-dehydrogenase (NADP(+), D-xylono-1,5-lactone-forming)
		EC3.2.1.23	EC	0.0787603	TRUE	beta-galactosidase
		EC5.1.3.3	EC	0.0443571	TRUE	aldose 1-epimerase
		EC5.3.1.5	EC	0.006861	TRUE	xylose isomerase
Wang et al. (ESRD)	8	C00116	Metabolite	NA	FALSE	Glycerol
		C06103	Metabolite	NA	FALSE	6-Hydroxyhexanoic acid
		EC1.1.1.2	EC	0.0001528	TRUE	alcohol dehydrogenase (NADP(+))
		EC2.7.1.30	EC	0.0116437	TRUE	glycerol kinase
		EC3.1.1.83	EC	0.0294566	TRUE	monoterpene epsilon-lactone hydrolase
		EC4.2.1.30	EC	0.0112488	TRUE	glycerol dehydratase
Wang et al. (ESRD)	9	C00135	Metabolite	NA	FALSE	L-Histidine
		C00785	Metabolite	NA	FALSE	Urocanate
		C03680	Metabolite	NA	FALSE	4-Imidazolone-5-propanoate
		EC1.1.1.23	EC	0.0192425	TRUE	histidinol dehydrogenase
		EC3.5.2.7	EC	0.0012146	TRUE	imidazolonepropionase
		EC4.2.1.49	EC	0.0006472	TRUE	urocanate hydratase
		EC4.3.1.3	EC	0.0001554	TRUE	histidine ammonia-lyase
		EC6.1.1.21	EC	0.0818091	TRUE	histidine--tRNA ligase
Wang et al. (ESRD)	10	C11638	Metabolite	NA	FALSE	3-Amino-2-oxopropyl phosphate
		C00627	Metabolite	NA	FALSE	Pyridoxine phosphate
		EC1.1.1.262	EC	0.0534713	TRUE	4-hydroxythreonine-4-phosphate dehydrogenase
		EC1.4.3.5	EC	0.003315	TRUE	pyridoxal 5'-phosphate synthase
		EC2.6.99.2	EC	0.0007355	TRUE	pyridoxine 5'-phosphate synthase
		EC2.7.1.35	EC	0.0979069	TRUE	pyridoxal kinase
Wang et al. (ESRD)	11	C11437	Metabolite	NA	FALSE	1-Deoxy-D-xylulose 5-phosphate
		C11434	Metabolite	NA	FALSE	2-C-Methyl-D-erythritol 4-phosphate
		C15809	Metabolite	NA	FALSE	Iminoglycine
		EC1.1.1.267	EC	0.0334971	TRUE	1-deoxy-D-xylulose-5-phosphate reductoisomerase
		EC2.2.1.7	EC	0.0281836	TRUE	1-deoxy-D-xylulose-5-phosphate synthase
		EC2.7.7.60	EC	0.0884026	TRUE	2-C-methyl-D-erythritol 4-phosphate cytidylyltransferase
		EC2.8.1.10	EC	0.0001998	TRUE	thiazole synthase
		EC4.1.99.19	EC	0.0262437	TRUE	2-iminoacetate synthase
Wang et al. (ESRD)	12	C00258	Metabolite	NA	FALSE	D-Glycerate
		C00160	Metabolite	NA	FALSE	Glycolate
		C00546	Metabolite	NA	FALSE	Methylglyoxal
		EC1.1.1.283	EC	0.0435836	TRUE	methylglyoxal reductase (NADPH)
		EC1.1.1.29	EC	0.0109704	TRUE	glycerate dehydrogenase
		EC1.1.1.79	EC	0.0740202	TRUE	glyoxylate reductase (NADP(+))
		EC1.1.1.81	EC	0.0110843	TRUE	hydroxypyruvate reductase
		EC3.8.1.2	EC	0.0515842	TRUE	(S)-2-haloacid dehalogenase
		EC4.4.1.5	EC	0.0368793	TRUE	lactoylglutathione lyase
Wang et al. (ESRD)	14	C11537	Metabolite	NA	FALSE	(2R)-3-Sulfolactate
		C00149	Metabolite	NA	FALSE	(S)-Malate
		EC1.1.1.37	EC	0.0001231	TRUE	malate dehydrogenase
		EC1.1.1.38	EC	0.0503857	TRUE	malate dehydrogenase (oxaloacetate-decarboxylating)
		EC1.1.1.40	EC	0.0000001	TRUE	malate dehydrogenase (oxaloacetate-decarboxylating) (NADP(+))
		EC3.1.3.71	EC	0.0489055	TRUE	2-phosphosulfolactate phosphatase
Wang et al. (ESRD)	15	C00199	Metabolite	NA	FALSE	D-Ribulose 5-phosphate
		C00345	Metabolite	NA	FALSE	6-Phospho-D-gluconate
		C00006	Metabolite	NA	FALSE	NADP+
		EC1.1.1.42	EC	0.0196171	TRUE	isocitrate dehydrogenase (NADP(+))
		EC1.1.1.44	EC	0.003237	TRUE	phosphogluconate dehydrogenase (NADP(+)-dependent, decarboxylating)
		EC1.1.1.49	EC	0.0013033	TRUE	glucose-6-phosphate dehydrogenase (NADP(+))
		EC1.8.1.7	EC	0.0936285	TRUE	glutathione-disulfide reductase
		EC2.7.1.12	EC	0.0863611	TRUE	gluconokinase
		EC2.7.1.16	EC	0.0336438	TRUE	ribulokinase
		EC3.1.1.31	EC	0.0040088	TRUE	6-phosphogluconolactonase
Wang et al. (ESRD)	16	C00232	Metabolite	NA	FALSE	Succinate semialdehyde
		C00091	Metabolite	NA	FALSE	Succinyl-CoA

		EC1.1.1.61	EC	0.0024204	TRUE	4-hydroxybutyrate dehydrogenase
		EC1.2.1.76	EC	0.0218723	TRUE	succinate-semialdehyde dehydrogenase (acetylating)
		EC1.2.7.3	EC	0.0068597	TRUE	2-oxoglutarate synthase
		EC5.4.99.2	EC	0.0088491	TRUE	methylmalonyl-CoA mutase
Wang et al. (ESRD)	17	C00233	Metabolite	NA	FALSE	4-Methyl-2-oxopentanoate
		C15972	Metabolite	NA	FALSE	Enzyme N6-(lipoyl)lysine
		C00141	Metabolite	NA	FALSE	3-Methyl-2-oxobutanoic acid
		C15980	Metabolite	NA	FALSE	(S)-2-Methylbutanoyl-CoA
		C15973	Metabolite	NA	FALSE	Enzyme N6-(dihydrolipoyl)lysine
		C22159	Metabolite	NA	FALSE	[Glycine cleavage system H]-N6-octanoyl-L-lysine
		EC1.1.1.85	EC	0.0102001	TRUE	3-isopropylmalate dehydrogenase
		EC1.2.4.4	EC	0.0083933	TRUE	3-methyl-2-oxobutanoate dehydrogenase (2-methylpropanoyl-transferring)
		EC1.2.7.7	EC	0.0368822	TRUE	3-methyl-2-oxobutanoate dehydrogenase (ferredoxin)
		EC1.3.8.1	EC	0.029919	TRUE	short-chain acyl-CoA dehydrogenase
		EC1.8.1.4	EC	0.0003511	TRUE	dihydrolipoyl dehydrogenase
		EC2.1.2.11	EC	0.0086436	TRUE	3-methyl-2-oxobutanoate hydroxymethyltransferase
		EC2.3.1.12	EC	0.0833308	TRUE	dihydrolipoyllysine-residue acetyltransferase
		EC2.3.1.181	EC	0.0001125	TRUE	lipoyl(octanoyl) transferase
		EC2.8.1.8	EC	0.0005155	TRUE	lipoyl synthase
Wang et al. (ESRD)	19	C03972	Metabolite	NA	FALSE	2,3,4,5-Tetrahydrodipicolinate
		C04462	Metabolite	NA	FALSE	N-Succinyl-2-L-amino-6-oxoheptanedioate
		C00666	Metabolite	NA	FALSE	LL-2,6-Diaminoheptanedioate
		EC1.17.1.8	EC	0.0009654	TRUE	4-hydroxy-tetrahydrodipicolinate reductase
		EC2.3.1.117	EC	0.0322356	TRUE	2,3,4,5-tetrahydropyridine-2,6-dicarboxylate N-succinyltransferase
		EC2.6.1.17	EC	0.0295176	TRUE	succinyldiaminopimelate transaminase
		EC2.6.1.83	EC	0.0000008	TRUE	LL-diaminopimelate aminotransferase
		EC3.5.1.18	EC	0.0059892	TRUE	succinyl-diaminopimelate desuccinylase
		EC3.5.1.47	EC	0.0341039	TRUE	N-acetyldiaminopimelate deacetylase
		EC5.1.1.7	EC	0.0327022	TRUE	diaminopimelate epimerase
Wang et al. (ESRD)	24	C00254	Metabolite	NA	FALSE	Prephenate
		EC1.3.1.12	EC	0.0289341	TRUE	prephenate dehydrogenase
		EC4.2.1.51	EC	0.0502756	TRUE	prephenate dehydratase
		EC4.2.1.91	EC	0.0556655	TRUE	arogenate dehydratase
Wang et al. (ESRD)	26	C02463	Metabolite	NA	FALSE	Precorrin 2
		C05778	Metabolite	NA	FALSE	Sirohydrochlorin
		C01051	Metabolite	NA	FALSE	Uroporphyrinogen III
		EC1.3.1.76	EC	0.0188292	TRUE	precorrin-2 dehydrogenase
		EC2.1.1.107	EC	0.060875	TRUE	uroporphyrinogen-III C-methyltransferase
		EC2.1.1.130	EC	0.0000032	TRUE	precorrin-2 C(20)-methyltransferase
		EC4.1.1.37	EC	0.064483	TRUE	uroporphyrinogen decarboxylase
		EC4.99.1.4	EC	0.0002904	TRUE	sirohydrochlorin ferrochelatase
Wang et al. (ESRD)	27	C00111	Metabolite	NA	FALSE	Glycerone phosphate
		C05840	Metabolite	NA	FALSE	Iminoaspartate
		EC1.4.3.16	EC	0.0016007	TRUE	L-aspartate oxidase
		EC2.5.1.72	EC	0.0002034	TRUE	quinolinate synthase
		EC2.7.1.121	EC	0.0139601	TRUE	phosphoenolpyruvate--glycerone phosphotransferase
		EC2.7.1.29	EC	0.0736325	TRUE	glycerone kinase
		EC4.1.2.40	EC	0.0170603	TRUE	tagatose-bisphosphate aldolase
		EC5.3.1.1	EC	0	TRUE	triose-phosphate isomerase
Wang et al. (ESRD)	29	C00064	Metabolite	NA	FALSE	L-Glutamine
		C00117	Metabolite	NA	FALSE	D-Ribose 5-phosphate
		EC1.4.7.1	EC	0	TRUE	glutamate synthase (ferredoxin)
		EC2.7.1.15	EC	0.0002112	TRUE	ribokinase
		EC2.7.6.1	EC	0.0008138	TRUE	ribose-phosphate diphosphokinase
		EC3.6.1.13	EC	0.0000009	TRUE	ADP-ribose diphosphatase
		EC4.3.3.6	EC	0	TRUE	pyridoxal 5'-phosphate synthase (glutamine hydrolyzing)
		EC6.1.1.18	EC	0.0005931	TRUE	glutamine--tRNA ligase
		EC6.3.1.2	EC	0.0032289	TRUE	glutamine synthetase
Wang et al. (ESRD)	30	C00870	Metabolite	NA	FALSE	4-Nitrophenol
		C00061	Metabolite	NA	FALSE	FMN
		C00378	Metabolite	NA	FALSE	Thiamine
		EC1.5.1.38	EC	0.0853902	TRUE	FMN reductase (NADPH)
		EC1.5.1.39	EC	0.0017566	TRUE	FMN reductase [NAD(P)H]
		EC2.7.1.26	EC	0.000868	TRUE	riboflavin kinase
		EC2.7.6.2	EC	0.0265151	TRUE	thiamine diphosphokinase
		EC2.7.7.2	EC	0.0008373	TRUE	FAD synthase
		EC3.1.3.1	EC	0.0007542	TRUE	alkaline phosphatase
		EC3.1.3.2	EC	0.1449444	FALSE	acid phosphatase
		EC3.1.8.1	EC	0.0021473	TRUE	aryldialkylphosphatase
Wang et al. (ESRD)	34	C00062	Metabolite	NA	FALSE	L-Arginine
		C00327	Metabolite	NA	FALSE	L-Citrulline
		C00077	Metabolite	NA	FALSE	L-Ornithine
		C15532	Metabolite	NA	FALSE	N-Acetyl-L-citrulline
		EC2.1.3.9	EC	0.077758	TRUE	N-acetylornithine carbamoyltransferase
		EC2.6.1.13	EC	0.0472936	TRUE	ornithine aminotransferase
		EC3.5.1.16	EC	0.0110068	TRUE	acetylornithine deacetylase
		EC3.5.3.1	EC	0.0239319	TRUE	arginase
		EC3.5.3.6	EC	0.0505194	TRUE	arginine deiminase
		EC4.3.2.1	EC	0.0056782	TRUE	argininosuccinate lyase
		EC6.3.4.5	EC	0.0750683	TRUE	argininosuccinate synthase
Wang et al. (ESRD)	36	C01063	Metabolite	NA	FALSE	Pimeloyl-CoA
		C01092	Metabolite	NA	FALSE	8-Amino-7-oxononanoate
		EC2.3.1.47	EC	0.0588412	TRUE	8-amino-7-oxononanoate synthase
		EC2.6.1.62	EC	0.0893766	TRUE	adenosylmethionine--8-amino-7-oxononanoate transaminase
		EC6.2.1.14	EC	0.0026619	TRUE	6-carboxyhexanoate--CoA ligase
Wang et al. (ESRD)	37	C00641	Metabolite	NA	FALSE	1,2-Diacyl-sn-glycerol
		C00416	Metabolite	NA	FALSE	Phosphatidate
		C01177	Metabolite	NA	FALSE	Inositol 1-phosphate
		EC2.3.1.51	EC	0.0149035	TRUE	1-acylglycerol-3-phosphate O-acyltransferase
		EC2.7.1.107	EC	0.082929	TRUE	diacylglycerol kinase (ATP)

		EC3.1.1.3	EC	0.0005783	TRUE	triacylglycerol lipase
		EC3.1.3.25	EC	0.0000931	TRUE	inositol-phosphate phosphatase
		EC3.1.3.4	EC	0.0600234	TRUE	phosphatidate phosphatase
		EC4.6.1.13	EC	0.0027144	TRUE	phosphatidylinositol diacylglycerol-lyase
Wang et al. (ESRD)	38	C00134	Metabolite	NA	FALSE	Putrescine
		C00315	Metabolite	NA	FALSE	Spermidine
		EC2.3.1.57	EC	0.0190643	TRUE	diamine N-acetyltransferase
		EC2.5.1.16	EC	0.2210627	FALSE	spermidine synthase
		EC3.5.1.53	EC	0.0318271	TRUE	N-carbamoylputrescine amidase
		EC3.5.3.11	EC	0.035426	TRUE	agmatinase
		EC4.1.1.96	EC	0.0010214	TRUE	carboxynorspermidine decarboxylase
Wang et al. (ESRD)	40	C00140	Metabolite	NA	FALSE	N-Acetyl-D-glucosamine
		EC2.4.1.280	EC	0.045516	TRUE	N,N'-diacetylchitobiose phosphorylase
		EC2.7.1.162	EC	0.0758786	TRUE	N-acetylhexosamine 1-kinase
		EC3.2.1.14	EC	0.0168406	TRUE	chitinase
Wang et al. (ESRD)	41	G13167	Metabolite	NA	FALSE	NA
		C00133	Metabolite	NA	FALSE	D-Alanine
		EC2.4.1.52	EC	0.0912361	TRUE	poly(glycerol-phosphate) alpha-glucosyltransferase
		EC6.1.1.13	EC	0.0013603	TRUE	D-alanine--poly(phosphoribitol) ligase
		EC6.3.2.4	EC	0.0327166	TRUE	D-alanine--D-alanine ligase
Wang et al. (ESRD)	42	C00106	Metabolite	NA	FALSE	Uracil
		C21028	Metabolite	NA	FALSE	(R)-5,6-Dihydrothymine
		C00299	Metabolite	NA	FALSE	Uridine
		EC1.3.1.2	EC	0.2442189	FALSE	dihydropyrimidine dehydrogenase (NADP(+))
		EC2.4.2.9	EC	0.0254023	TRUE	uracil phosphoribosyltransferase
		EC3.2.2.3	EC	0.0861802	TRUE	uridine nucleosidase
		EC3.5.2.2	EC	0.0003186	TRUE	dihydropyrimidinase
		EC3.5.4.5	EC	0.0000754	TRUE	cytidine deaminase
		EC4.2.1.70	EC	0.0763505	TRUE	pseudouridylate synthase
Wang et al. (ESRD)	43	C00074	Metabolite	NA	FALSE	Phosphoenolpyruvate
		C04691	Metabolite	NA	FALSE	2-Dehydro-3-deoxy-D-arabino-heptonate 7-phosphate
		EC2.5.1.54	EC	0.0227864	TRUE	3-deoxy-7-phosphoheptulonate synthase
		EC2.7.9.2	EC	0.0337732	TRUE	pyruvate, water dikinase
		EC4.1.1.31	EC	0.0553958	TRUE	phosphoenolpyruvate carboxylase
		EC4.1.1.32	EC	0.0196531	TRUE	phosphoenolpyruvate carboxykinase (GTP)
		EC4.1.1.49	EC	0.0231634	TRUE	phosphoenolpyruvate carboxykinase (ATP)
		EC4.2.3.4	EC	0.0003653	TRUE	3-dehydroquinate synthase
Wang et al. (ESRD)	45	C19972	Metabolite	NA	FALSE	2,4-Bis(acetamido)-2,4,6-trideoxy-beta-L-altropyranose
		C20082	Metabolite	NA	FALSE	Pseudaminic acid
		EC2.5.1.97	EC	0.0616637	TRUE	pseudaminic acid synthase
		EC2.7.7.81	EC	0.0374947	TRUE	pseudaminic acid cytidylyltransferase
		EC3.6.1.57	EC	0.0963297	TRUE	UDP-2,4-diacetamido-2,4,6-trideoxy-beta-L-altropyranose hydrolase
Wang et al. (ESRD)	46	C04188	Metabolite	NA	FALSE	S-Methyl-5-thio-D-ribose 1-phosphate
		C19787	Metabolite	NA	FALSE	5'-S-Methyl-5'-thioinosine
		EC2.7.1.100	EC	0.0004615	TRUE	S-methyl-5-thioribose kinase
		EC3.5.4.31	EC	0.0701095	TRUE	S-methyl-5'-thioadenosine deaminase
		EC5.3.1.23	EC	0.0775393	TRUE	S-methyl-5-thioribose-1-phosphate isomerase
		EC2.4.2.44	EC	NA	FALSE	S-methyl-5'-thioinosine phosphorylase
Wang et al. (ESRD)	49	C04556	Metabolite	NA	FALSE	4-Amino-2-methyl-5-(phosphooxymethyl)pyrimidine
		C04751	Metabolite	NA	FALSE	1-(5-Phospho-D-ribosyl)-5-amino-4-imidazolecarboxylate
		C03373	Metabolite	NA	FALSE	Aminoimidazole ribotide
		C15667	Metabolite	NA	FALSE	5-Carboxyamino-1-(5-phospho-D-ribosyl)imidazole
		EC2.7.1.49	EC	0.0008382	TRUE	hydroxymethylpyrimidine kinase
		EC2.7.4.7	EC	0.0065149	TRUE	phosphooxymethylpyrimidine kinase
		EC4.1.99.17	EC	0.0033584	TRUE	phosphomethylpyrimidine synthase
		EC5.4.99.18	EC	0.0119016	TRUE	5-(carboxyamino)imidazole ribonucleotide mutase
		EC6.3.2.6	EC	0.0403274	TRUE	phosphoribosylaminoimidazolesuccinocarboxamide synthase
		EC6.3.3.1	EC	0.0528415	TRUE	phosphoribosylformylglycinamidine cyclo-ligase
		EC6.3.4.18	EC	0.0076346	TRUE	5-(carboxyamino)imidazole ribonucleotide synthase
Wang et al. (ESRD)	51	C00245	Metabolite	NA	FALSE	Taurine
		C00099	Metabolite	NA	FALSE	beta-Alanine
		C00334	Metabolite	NA	FALSE	4-Aminobutanoate
		EC3.5.1.24	EC	0.0939251	TRUE	choloylglycine hydrolase
		EC3.5.1.94	EC	0.0905836	TRUE	gamma-glutamyl-gamma-aminobutyrate hydrolase
		EC4.1.1.11	EC	0.000978	TRUE	aspartate 1-decarboxylase
		EC4.1.1.15	EC	0.0007686	TRUE	glutamate decarboxylase
		EC6.3.2.1	EC	0.0125214	TRUE	pantoate--beta-alanine ligase (AMP-forming)

Supplementary Table S7: Modules significant overlaps with KEGG pathways

Dataset	Module ID	KEGG pathway code	KEGG pathway description	Module size	Pathway size	No. of EC nodes in overlap	No. of metabolite nodes in overlap	Hypergeometric test - FDR
Franzosa et al. (CD)	1	map00220	Arginine biosynthesis	8	38	1	2	0.0000416
Franzosa et al. (CD)	1	map00330	Arginine and proline metabolism	8	119	2	2	0.0000402
Franzosa et al. (CD)	2	map00030	Pentose phosphate pathway	14	82	3	2	0.0000055
Franzosa et al. (CD)	2	map00230	Purine metabolism	14	170	1	3	0.0013823
Franzosa et al. (CD)	2	map00240	Pyrimidine metabolism	14	113	1	2	0.0042623
Franzosa et al. (CD)	2	map00250	Alanine, aspartate and glutamate metabolism	14	49	2	1	0.0004182
Franzosa et al. (CD)	2	map00750	Vitamin B6 metabolism	14	42	2	4	0
Franzosa et al. (CD)	2	map01230	Biosynthesis of amino acids	14	113	0	3	0.0042623
Franzosa et al. (CD)	2	map01240	Biosynthesis of cofactors	14	297	0	4	0.0095605
Franzosa et al. (CD)	3	map00270	Cysteine and methionine metabolism	7	129	3	4	0
Franzosa et al. (CD)	5	map00280	Valine, leucine and isoleucine degradation	7	65	1	4	0
Franzosa et al. (CD)	5	map00290	Valine, leucine and isoleucine biosynthesis	7	33	2	5	0
Franzosa et al. (CD)	5	map00770	Pantothenate and CoA biosynthesis	7	47	1	2	0.0000475
Franzosa et al. (CD)	5	map00966	Glucosinolate biosynthesis	7	11	1	4	0
Franzosa et al. (CD)	5	map00970	Aminoacyl-tRNA biosynthesis	7	61	0	3	0.0000967
Franzosa et al. (CD)	5	map01060	Biosynthesis of plant secondary metabolites	7	96	0	3	0.0003311
Franzosa et al. (CD)	5	map01210	2-Oxocarboxylic acid metabolism	7	63	0	5	0
Franzosa et al. (CD)	5	map01230	Biosynthesis of amino acids	7	113	0	5	0.0000005
Franzosa et al. (CD)	5	map02010	ABC transporters	7	76	0	3	0.0001766
Franzosa et al. (CD)	5	map04974	Protein digestion and absorption	7	27	0	3	0.000011
Franzosa et al. (CD)	5	map04978	Mineral absorption	7	17	0	3	0.0000034
Franzosa et al. (CD)	5	map05230	Central carbon metabolism in cancer	7	25	0	3	0.0000092
Franzosa et al. (CD)	7	map00230	Purine metabolism	26	170	12	9	0
Franzosa et al. (CD)	7	map00240	Pyrimidine metabolism	26	113	2	2	0.0033751
Franzosa et al. (CD)	7	map00760	Nicotinate and nicotinamide metabolism	26	93	2	1	0.0139411
Franzosa et al. (CD)	7	map01060	Biosynthesis of plant secondary metabolites	26	96	0	5	0.0001915
Franzosa et al. (CD)	7	map01065	Biosynthesis of alkaloids derived from histidine and purine	26	27	0	4	0.000021
Franzosa et al. (CD)	7	map01232	Nucleotide metabolism	26	57	0	10	0
Franzosa et al. (CD)	7	map02010	ABC transporters	26	76	0	4	0.0008103
Franzosa et al. (CD)	9	map00620	Pyruvate metabolism	5	75	2	1	0.0000551
Franzosa et al. (CD)	9	map00640	Propanoate metabolism	5	79	2	3	0
Franzosa et al. (CD)	11	map00565	Ether lipid metabolism	6	26	2	1	0.0000064
Franzosa et al. (CD)	11	map00591	Linoleic acid metabolism	6	8	1	2	0.0000002
Franzosa et al. (CD)	11	map01040	Biosynthesis of unsaturated fatty acids	6	31	0	3	0.0000101
Franzosa et al. (CD)	12	map00120	Primary bile acid biosynthesis	6	19	1	5	0
Franzosa et al. (CD)	12	map00121	Secondary bile acid biosynthesis	6	9	1	4	0
Franzosa et al. (CD)	12	map04976	Bile secretion	6	34	0	4	0.0000001
Franzosa et al. (CD)	13	map00730	Thiamine metabolism	8	48	2	1	0.0000756
Franzosa et al. (CD)	13	map00740	Riboflavin metabolism	8	47	3	2	0
Franzosa et al. (CD)	13	map01240	Biosynthesis of cofactors	8	297	0	3	0.0110714
Franzosa et al. (CD)	13	map04977	Vitamin digestion and absorption	8	29	0	3	0.000021
Franzosa et al. (CD)	15	map00260	Glycine, serine and threonine metabolism	5	102	2	3	0
Franzosa et al. (CD)	15	map00330	Arginine and proline metabolism	5	119	1	2	0.000193
Franzosa et al. (CD)	17	map00350	Tyrosine metabolism	11	80	3	1	0.0000379
Franzosa et al. (CD)	17	map00360	Phenylalanine metabolism	11	78	4	2	0
Franzosa et al. (CD)	17	map00400	Phenylalanine, tyrosine and tryptophan biosynthesis	11	62	3	2	0.0000005
Franzosa et al. (CD)	17	map00401	Novobiocin biosynthesis	11	21	2	1	0.0000216
Franzosa et al. (CD)	17	map00470	D-Amino acid metabolism	11	84	2	2	0.000043
Franzosa et al. (CD)	17	map00960	Tropane, piperidine and pyridine alkaloid biosynthesis	11	32	2	2	0.0000015
Franzosa et al. (CD)	21	map00040	Pentose and glucuronate interconversions	18	109	2	1	0.0078875
Franzosa et al. (CD)	21	map00240	Pyrimidine metabolism	18	113	4	2	0.0000055
Franzosa et al. (CD)	21	map00500	Starch and sucrose metabolism	18	91	4	1	0.0000281
Franzosa et al. (CD)	21	map00670	One carbon pool by folate	18	29	4	2	0
Franzosa et al. (CD)	21	map00760	Nicotinate and nicotinamide metabolism	18	93	3	1	0.0004182
Franzosa et al. (CD)	21	map01240	Biosynthesis of cofactors	18	297	0	5	0.0043585
Franzosa et al. (CD)	21	map01523	Antifolate resistance	18	13	0	3	0.0000221
Franzosa et al. (CD)	22	map00010	Glycolysis / Gluconeogenesis	10	67	2	1	0.0003711
Franzosa et al. (CD)	22	map00051	Fructose and mannose metabolism	10	116	3	2	0.0000045
Franzosa et al. (CD)	22	map00500	Starch and sucrose metabolism	10	91	3	2	0.0000015
Franzosa et al. (CD)	22	map00520	Amino sugar and nucleotide sugar metabolism	10	215	4	2	0.0000037
Franzosa et al. (CD)	22	map00541	O-Antigen nucleotide sugar biosynthesis	10	118	1	2	0.0017899
Franzosa et al. (CD)	26	map00520	Amino sugar and nucleotide sugar metabolism	4	215	3	1	0.0000087
Franzosa et al. (UC)	1	map00260	Glycine, serine and threonine metabolism	8	102	2	1	0.0006315
Franzosa et al. (UC)	1	map00270	Cysteine and methionine metabolism	8	129	2	1	0.0012005
Franzosa et al. (UC)	1	map00310	Lysine degradation	8	65	2	1	0.0001992
Franzosa et al. (UC)	1	map00470	D-Amino acid metabolism	8	84	3	3	0
Franzosa et al. (UC)	2	map00270	Cysteine and methionine metabolism	21	129	9	3	0
Franzosa et al. (UC)	2	map00350	Tyrosine metabolism	21	80	4	1	0.0000342
Franzosa et al. (UC)	2	map00360	Phenylalanine metabolism	21	78	4	0	0.0004432
Franzosa et al. (UC)	2	map00400	Phenylalanine, tyrosine and tryptophan biosynthesis	21	62	4	2	0.0000004
Franzosa et al. (UC)	2	map00401	Novobiocin biosynthesis	21	21	4	1	0.0000001
Franzosa et al. (UC)	2	map00450	Selenocompound metabolism	21	31	5	1	0
Franzosa et al. (UC)	2	map00950	Isoquinoline alkaloid biosynthesis	21	14	2	1	0.0000438
Franzosa et al. (UC)	2	map00960	Tropane, piperidine and pyridine alkaloid biosynthesis	21	32	3	0	0.0004564
Franzosa et al. (UC)	2	map01230	Biosynthesis of amino acids	21	113	0	4	0.0015885
Franzosa et al. (UC)	5	map00010	Glycolysis / Gluconeogenesis	11	67	3	1	0.0000204
Franzosa et al. (UC)	5	map00620	Pyruvate metabolism	11	75	7	1	0
Franzosa et al. (UC)	5	map00640	Propanoate metabolism	11	79	7	2	0
Franzosa et al. (UC)	6	map00230	Purine metabolism	30	170	10	8	0
Franzosa et al. (UC)	6	map00240	Pyrimidine metabolism	30	113	10	3	0
Franzosa et al. (UC)	6	map00983	Drug metabolism - other enzymes	30	58	4	1	0.0000438
Franzosa et al. (UC)	6	map01232	Nucleotide metabolism	30	57	0	10	0

Franzosa et al. (UC)	6	map02010	ABC transporters	30	76	0	5	0.0001522
Franzosa et al. (UC)	8	map00564	Glycerophospholipid metabolism	7	81	4	1	0.0000001
Franzosa et al. (UC)	8	map00591	Linoleic acid metabolism	7	8	1	2	0.0000003
Franzosa et al. (UC)	14	map00010	Glycolysis / Gluconeogenesis	14	67	3	2	0.000002
Franzosa et al. (UC)	14	map00020	Citrate cycle (TCA cycle)	14	39	5	2	0
Franzosa et al. (UC)	14	map00260	Glycine, serine and threonine metabolism	14	102	2	1	0.0033262
Franzosa et al. (UC)	14	map00280	Valine, leucine and isoleucine degradation	14	65	1	2	0.0009869
Franzosa et al. (UC)	14	map00310	Lysine degradation	14	65	2	2	0.0000476
Franzosa et al. (UC)	14	map00620	Pyruvate metabolism	14	75	3	2	0.0000033
Franzosa et al. (UC)	14	map00640	Propanoate metabolism	14	79	1	2	0.001666
Franzosa et al. (UC)	14	map00785	Lipoic acid metabolism	14	48	9	5	0
Franzosa et al. (UC)	14	map01240	Biosynthesis of cofactors	14	297	0	4	0.0098416
Franzosa et al. (UC)	18	map00010	Glycolysis / Gluconeogenesis	13	67	4	2	0
Franzosa et al. (UC)	18	map00030	Pentose phosphate pathway	13	82	3	2	0.0000033
Franzosa et al. (UC)	18	map00051	Fructose and mannose metabolism	13	116	6	2	0
Franzosa et al. (UC)	18	map00500	Starch and sucrose metabolism	13	91	2	1	0.0019717
Franzosa et al. (UC)	18	map00520	Amino sugar and nucleotide sugar metabolism	13	215	3	2	0.0002541
Franzosa et al. (UC)	18	map00541	O-Antigen nucleotide sugar biosynthesis	13	118	1	2	0.0039508
Franzosa et al. (UC)	23	map00260	Glycine, serine and threonine metabolism	12	102	2	1	0.0021132
Franzosa et al. (UC)	23	map00361	Chlorocyclohexane and chlorobenzene degradation	12	81	2	1	0.0011536
Franzosa et al. (UC)	23	map00625	Chloroalkane and chloroalkene degradation	12	45	2	1	0.00024
Franzosa et al. (UC)	23	map00630	Glyoxylate and dicarboxylate metabolism	12	86	7	3	0
Franzosa et al. (UC)	24	map00250	Alanine, aspartate and glutamate metabolism	15	49	2	1	0.0005533
Franzosa et al. (UC)	24	map00310	Lysine degradation	15	65	2	1	0.0012005
Franzosa et al. (UC)	24	map00330	Arginine and proline metabolism	15	119	3	1	0.0005421
Franzosa et al. (UC)	24	map00410	beta-Alanine metabolism	15	39	4	3	0
Franzosa et al. (UC)	24	map00640	Propanoate metabolism	15	79	2	2	0.0001341
Franzosa et al. (UC)	24	map00650	Butanoate metabolism	15	88	2	1	0.0027201
Franzosa et al. (UC)	41	map00071	Fatty acid degradation	13	47	3	3	0
Franzosa et al. (UC)	41	map00310	Lysine degradation	13	65	1	2	0.0007883
Franzosa et al. (UC)	41	map00362	Benzoate degradation	13	118	2	1	0.0039508
Franzosa et al. (UC)	41	map00650	Butanoate metabolism	13	88	5	2	0
Franzosa et al. (UC)	41	map00720	Carbon fixation pathways in prokaryotes	13	83	1	2	0.0015492
Franzosa et al. (UC)	41	map01200	Carbon metabolism	13	92	0	3	0.0020222
Franzosa et al. (UC)	41	map01212	Fatty acid metabolism	13	64	0	3	0.0007633
Franzosa et al. (UC)	45	map00130	Ubiquinone and other terpenoid-quinone biosynthesis	7	84	2	2	0.0000063
Franzosa et al. (UC)	45	map01053	Biosynthesis of siderophore group nonribosomal peptides	7	19	2	2	0
Franzosa et al. (UC)	46	map00061	Fatty acid biosynthesis	12	50	1	2	0.000312
Franzosa et al. (UC)	46	map00071	Fatty acid degradation	12	47	1	2	0.0002651
Franzosa et al. (UC)	46	map00540	Lipopolysaccharide biosynthesis	12	77	3	3	0
Franzosa et al. (UC)	46	map00770	Pantothenate and CoA biosynthesis	12	47	3	2	0.0000002
Franzosa et al. (UC)	48	map00240	Pyrimidine metabolism	10	113	3	1	0.0000917
Franzosa et al. (UC)	48	map00670	One carbon pool by folate	10	29	6	2	0
Franzosa et al. (UC)	48	map00720	Carbon fixation pathways in prokaryotes	10	83	2	2	0.0000296
Franzosa et al. (UC)	61	map00910	Nitrogen metabolism	6	41	2	2	0.0000002
Jeffery et al. (IBS)	2	map00220	Arginine biosynthesis	25	38	3	2	0.000003
Jeffery et al. (IBS)	2	map00250	Alanine, aspartate and glutamate metabolism	25	49	6	1	0
Jeffery et al. (IBS)	2	map00260	Glycine, serine and threonine metabolism	25	102	6	2	0.0000001
Jeffery et al. (IBS)	2	map00270	Cysteine and methionine metabolism	25	129	2	1	0.030691
Jeffery et al. (IBS)	2	map00300	Lysine biosynthesis	25	59	3	2	0.0000218
Jeffery et al. (IBS)	2	map00310	Lysine degradation	25	65	2	1	0.0051024
Jeffery et al. (IBS)	2	map00330	Arginine and proline metabolism	25	119	2	1	0.0251289
Jeffery et al. (IBS)	2	map00470	D-Amino acid metabolism	25	84	3	5	0
Jeffery et al. (IBS)	2	map00630	Glyoxylate and dicarboxylate metabolism	25	86	2	2	0.001212
Jeffery et al. (IBS)	2	map00680	Methane metabolism	25	156	2	1	0.0489446
Jeffery et al. (IBS)	2	map00910	Nitrogen metabolism	25	41	2	1	0.0015025
Jeffery et al. (IBS)	2	map00970	Aminoacyl-tRNA biosynthesis	25	61	0	5	0.0000248
Jeffery et al. (IBS)	2	map00997	Biosynthesis of various other secondary metabolites	25	46	0	3	0.0020471
Jeffery et al. (IBS)	2	map01060	Biosynthesis of plant secondary metabolites	25	96	0	5	0.0001732
Jeffery et al. (IBS)	2	map01230	Biosynthesis of amino acids	25	113	0	6	0.0000353
Jeffery et al. (IBS)	2	map02010	ABC transporters	25	76	0	4	0.0007763
Jeffery et al. (IBS)	2	map04974	Protein digestion and absorption	25	27	0	5	0.0000006
Jeffery et al. (IBS)	2	map04978	Mineral absorption	25	17	0	3	0.0001318
Jeffery et al. (IBS)	2	map05230	Central carbon metabolism in cancer	25	25	0	4	0.0000144
Jeffery et al. (IBS)	4	map00230	Purine metabolism	18	170	6	3	0
Jeffery et al. (IBS)	4	map00760	Nicotinate and nicotinamide metabolism	18	93	7	3	0
Jeffery et al. (IBS)	4	map01232	Nucleotide metabolism	18	57	0	3	0.0014778
Jeffery et al. (IBS)	16	map00071	Fatty acid degradation	12	47	2	3	0.0000002
Jeffery et al. (IBS)	16	map00280	Valine, leucine and isoleucine degradation	12	65	2	1	0.000644
Jeffery et al. (IBS)	16	map00310	Lysine degradation	12	65	1	3	0.0000272
Jeffery et al. (IBS)	16	map00362	Benzoate degradation	12	118	1	2	0.0032695
Jeffery et al. (IBS)	16	map00630	Glyoxylate and dicarboxylate metabolism	12	86	1	2	0.001415
Jeffery et al. (IBS)	16	map00650	Butanoate metabolism	12	88	7	4	0
Jeffery et al. (IBS)	16	map01200	Carbon metabolism	12	92	0	3	0.0016749
Jeffery et al. (IBS)	16	map01212	Fatty acid metabolism	12	64	0	3	0.0006187
Jeffery et al. (IBS)	33	map00240	Pyrimidine metabolism	16	113	10	6	0
Jeffery et al. (IBS)	33	map00410	beta-Alanine metabolism	16	39	2	1	0.000367
Jeffery et al. (IBS)	33	map00770	Pantothenate and CoA biosynthesis	16	47	2	1	0.0006143
Jeffery et al. (IBS)	33	map00983	Drug metabolism - other enzymes	16	58	4	0	0.0000547
Jeffery et al. (IBS)	33	map01232	Nucleotide metabolism	16	57	0	5	0.0000022
Jeffery et al. (IBS)	38	map00730	Thiamine metabolism	15	48	4	2	0
Jeffery et al. (IBS)	38	map00740	Riboflavin metabolism	15	47	5	3	0
Jeffery et al. (IBS)	38	map01240	Biosynthesis of cofactors	15	297	0	5	0.0020407
Jeffery et al. (IBS)	38	map04977	Vitamin digestion and absorption	15	29	0	3	0.0001381
Jeffery et al. (IBS)	40	map00860	Porphyrin metabolism	5	186	3	2	0.0000003
Jeffery et al. (IBS)	47	map00790	Folate biosynthesis	5	97	3	2	0
Jeffery et al. (IBS)	48	map00730	Thiamine metabolism	5	48	3	2	0
Jeffery et al. (IBS)	51	map00780	Biotin metabolism	7	34	4	3	0
Jeffery et al. (IBS)	51	map01240	Biosynthesis of cofactors	7	297	0	3	0.0076235
Jeffery et al. (IBS)	53	map00040	Pentose and glucuronate interconversions	25	109	1	2	0.0200421

Jeffery et al. (IBS)	53	map00052	Galactose metabolism	25	73	3	4	0.0000002
Jeffery et al. (IBS)	53	map00500	Starch and sucrose metabolism	25	91	15	6	0
Jeffery et al. (IBS)	53	map00520	Amino sugar and nucleotide sugar metabolism	25	215	4	3	0.0001362
Jeffery et al. (IBS)	53	map00541	O-Antigen nucleotide sugar biosynthesis	25	118	3	3	0.0000432
Jeffery et al. (IBS)	53	map00561	Glycerolipid metabolism	25	56	1	2	0.0034615
Jeffery et al. (IBS)	53	map01250	Biosynthesis of nucleotide sugars	25	139	0	3	0.0368771
Jeffery et al. (IBS)	59	map00900	Terpenoid backbone biosynthesis	5	50	4	1	0
Jeffery et al. (IBS)	60	map00860	Porphyrin metabolism	8	186	5	3	0
Jeffery et al. (IBS)	68	map00220	Arginine biosynthesis	5	38	3	2	0
Jeffery et al. (IBS)	68	map00791	Atrazine degradation	5	18	3	2	0
Wang et al. (ESRD)	6	map00260	Glycine, serine and threonine metabolism	6	102	4	2	0
Wang et al. (ESRD)	7	map00040	Pentose and glucuronate interconversions	8	109	3	2	0.0000008
Wang et al. (ESRD)	7	map00051	Fructose and mannose metabolism	8	116	2	1	0.0008585
Wang et al. (ESRD)	7	map00052	Galactose metabolism	8	73	2	1	0.0002393
Wang et al. (ESRD)	8	map00561	Glycerolipid metabolism	6	56	3	1	0.0000007
Wang et al. (ESRD)	8	map00930	Caprolactam degradation	6	20	2	1	0.0000031
Wang et al. (ESRD)	9	map00340	Histidine metabolism	8	44	4	3	0
Wang et al. (ESRD)	10	map00750	Vitamin B6 metabolism	6	42	4	2	0
Wang et al. (ESRD)	11	map00730	Thiamine metabolism	8	48	3	2	0
Wang et al. (ESRD)	11	map00900	Terpenoid backbone biosynthesis	8	50	3	2	0
Wang et al. (ESRD)	12	map00260	Glycine, serine and threonine metabolism	9	102	2	2	0.0000395
Wang et al. (ESRD)	12	map00620	Pyruvate metabolism	9	75	3	1	0.0000134
Wang et al. (ESRD)	12	map00630	Glyoxylate and dicarboxylate metabolism	9	86	3	2	0.0000006
Wang et al. (ESRD)	14	map00620	Pyruvate metabolism	6	75	3	1	0.0000022
Wang et al. (ESRD)	14	map00680	Methane metabolism	6	156	2	2	0.0000288
Wang et al. (ESRD)	14	map00710	Carbon fixation in photosynthetic organisms	6	35	2	1	0.0000139
Wang et al. (ESRD)	15	map00030	Pentose phosphate pathway	10	82	4	2	0
Wang et al. (ESRD)	15	map00480	Glutathione metabolism	10	47	4	1	0.0000001
Wang et al. (ESRD)	16	map00650	Butanoate metabolism	6	88	2	2	0.0000039
Wang et al. (ESRD)	16	map00720	Carbon fixation pathways in prokaryotes	6	83	3	2	0
Wang et al. (ESRD)	17	map00010	Glycolysis / Gluconeogenesis	15	67	2	2	0.000069
Wang et al. (ESRD)	17	map00020	Citrate cycle (TCA cycle)	15	39	2	2	0.0000105
Wang et al. (ESRD)	17	map00280	Valine, leucine and isoleucine degradation	15	65	4	5	0
Wang et al. (ESRD)	17	map00290	Valine, leucine and isoleucine biosynthesis	15	33	1	2	0.0001782
Wang et al. (ESRD)	17	map00310	Lysine degradation	15	65	1	2	0.0011322
Wang et al. (ESRD)	17	map00620	Pyruvate metabolism	15	75	2	2	0.0001023
Wang et al. (ESRD)	17	map00640	Propanoate metabolism	15	79	3	2	0.0000066
Wang et al. (ESRD)	17	map00785	Lipoic acid metabolism	15	48	5	6	0
Wang et al. (ESRD)	17	map01210	2-Oxocarboxylic acid metabolism	15	63	0	3	0.0010414
Wang et al. (ESRD)	17	map01240	Biosynthesis of cofactors	15	297	0	4	0.012395
Wang et al. (ESRD)	19	map00300	Lysine biosynthesis	10	59	7	3	0
Wang et al. (ESRD)	19	map01230	Biosynthesis of amino acids	10	113	0	3	0.0015409
Wang et al. (ESRD)	24	map00400	Phenylalanine, tyrosine and tryptophan biosynthesis	4	62	3	1	0.0000001
Wang et al. (ESRD)	26	map00860	Porphyrin metabolism	8	186	5	3	0
Wang et al. (ESRD)	26	map01240	Biosynthesis of cofactors	8	297	0	3	0.0112675
Wang et al. (ESRD)	27	map00561	Glycerolipid metabolism	8	56	2	1	0.0001198
Wang et al. (ESRD)	27	map00760	Nicotinate and nicotinamide metabolism	8	93	2	2	0.0000172
Wang et al. (ESRD)	29	map00030	Pentose phosphate pathway	9	82	2	1	0.0004719
Wang et al. (ESRD)	29	map00230	Purine metabolism	9	170	2	2	0.0002285
Wang et al. (ESRD)	29	map00630	Glyoxylate and dicarboxylate metabolism	9	86	2	1	0.0005343
Wang et al. (ESRD)	29	map00750	Vitamin B6 metabolism	9	42	1	2	0.0000778
Wang et al. (ESRD)	29	map00910	Nitrogen metabolism	9	41	2	1	0.0000746
Wang et al. (ESRD)	30	map00730	Thiamine metabolism	11	48	3	1	0.0000064
Wang et al. (ESRD)	30	map00740	Riboflavin metabolism	11	47	5	1	0
Wang et al. (ESRD)	34	map00220	Arginine biosynthesis	11	38	6	4	0
Wang et al. (ESRD)	34	map00330	Arginine and proline metabolism	11	119	2	2	0.0001517
Wang et al. (ESRD)	34	map01230	Biosynthesis of amino acids	11	113	0	4	0.0001251
Wang et al. (ESRD)	36	map00780	Biotin metabolism	5	34	3	2	0
Wang et al. (ESRD)	37	map00561	Glycerolipid metabolism	9	56	4	2	0
Wang et al. (ESRD)	37	map00562	Inositol phosphate metabolism	9	51	2	2	0.0000035
Wang et al. (ESRD)	37	map00564	Glycerophospholipid metabolism	9	81	3	2	0.0000005
Wang et al. (ESRD)	37	map04070	Phosphatidylinositol signaling system	9	15	0	3	0.0000049
Wang et al. (ESRD)	38	map00330	Arginine and proline metabolism	7	119	5	2	0
Wang et al. (ESRD)	38	map00480	Glutathione metabolism	7	47	1	2	0.0000498
Wang et al. (ESRD)	40	map00520	Amino sugar and nucleotide sugar metabolism	4	215	2	1	0.0004356
Wang et al. (ESRD)	41	map00470	D-Amino acid metabolism	5	84	2	1	0.0000778
Wang et al. (ESRD)	41	map00552	Teichoic acid biosynthesis	5	32	2	1	0.0000062
Wang et al. (ESRD)	42	map00240	Pyrimidine metabolism	9	113	6	3	0
Wang et al. (ESRD)	42	map00410	beta-Alanine metabolism	9	39	2	1	0.0000655
Wang et al. (ESRD)	42	map00770	Pantothenate and CoA biosynthesis	9	47	2	1	0.0001062
Wang et al. (ESRD)	42	map00983	Drug metabolism - other enzymes	9	58	3	0	0.0001852
Wang et al. (ESRD)	43	map00010	Glycolysis / Gluconeogenesis	8	67	3	1	0.0000056
Wang et al. (ESRD)	43	map00020	Citrate cycle (TCA cycle)	8	39	2	1	0.0000454
Wang et al. (ESRD)	43	map00400	Phenylalanine, tyrosine and tryptophan biosynthesis	8	62	2	2	0.0000042
Wang et al. (ESRD)	43	map00620	Pyruvate metabolism	8	75	4	1	0.0000002
Wang et al. (ESRD)	43	map00680	Methane metabolism	8	156	2	1	0.0018833
Wang et al. (ESRD)	43	map00710	Carbon fixation in photosynthetic organisms	8	35	2	1	0.0000341
Wang et al. (ESRD)	43	map00720	Carbon fixation pathways in prokaryotes	8	83	2	1	0.0003446
Wang et al. (ESRD)	45	map00520	Amino sugar and nucleotide sugar metabolism	5	215	3	2	0.0000005
Wang et al. (ESRD)	45	map00541	O-Antigen nucleotide sugar biosynthesis	5	118	3	2	0
Wang et al. (ESRD)	46	map00270	Cysteine and methionine metabolism	6	129	4	2	0
Wang et al. (ESRD)	49	map00230	Purine metabolism	11	170	4	3	0.0000001
Wang et al. (ESRD)	49	map00730	Thiamine metabolism	11	48	3	2	0.0000001
Wang et al. (ESRD)	51	map00410	beta-Alanine metabolism	8	39	3	2	0
Wang et al. (ESRD)	51	map00770	Pantothenate and CoA biosynthesis	8	47	2	1	0.000075
Wang et al. (ESRD)	51	map04080	Neuroactive ligand-receptor interaction	8	27	0	3	0.0000171

* Only overlaps with significant modules are shown.

Supplementary Table S8: Pathway-disease associations (KEGG pathways) using multiple analysis approaches

Dataset	Test name	KEGG pathway ID	Pathway name	P-value	FDR	Direction
Wang et al. (ESRD)	Pathway-level DA	map00730	Thiamine metabolism	1.4E-10	6.97E-08	Under-represented in disease
Wang et al. (ESRD)	Pathway-level DA	map00740	Riboflavin metabolism	5.62E-08	1.39E-05	Under-represented in disease
Wang et al. (ESRD)	Pathway-level DA	map00930	Caprolactam degradation	5.7E-07	9.43E-05	Under-represented in disease
Wang et al. (ESRD)	Pathway-level DA	map00562	Inositol phosphate metabolism	3.66E-06	0.000453	Under-represented in disease
Wang et al. (ESRD)	Pathway-level DA	map00250	Alanine, aspartate and glutamate metabolism	1.24E-05	0.001226	Under-represented in disease
Wang et al. (ESRD)	Pathway-level DA	map00332	Carbapenem biosynthesis	2.81E-05	0.002319	Under-represented in disease
Wang et al. (ESRD)	Pathway-level DA	map00470	D-Amino acid metabolism	6.52E-05	0.00404	Under-represented in disease
Wang et al. (ESRD)	Pathway-level DA	map01055	Biosynthesis of vancomycin group antibiotics	0.00022	0.008388	Under-represented in disease
Wang et al. (ESRD)	Pathway-level DA	map00710	Carbon fixation in photosynthetic organisms	0.000269	0.008893	Under-represented in disease
Wang et al. (ESRD)	Pathway-level DA	map00790	Folate biosynthesis	0.000268	0.008893	Under-represented in disease
Wang et al. (ESRD)	Pathway-level DA	map00650	Butanoate metabolism	3.14E-04	0.009745	Over-represented in disease
Wang et al. (ESRD)	Pathway-level DA	map00410	beta-Alanine metabolism	3.58E-04	0.009869	Under-represented in disease
Wang et al. (ESRD)	Pathway-level DA	map00230	Purine metabolism	0.000473	0.01235	Under-represented in disease
Wang et al. (ESRD)	Pathway-level DA	map00300	Lysine biosynthesis	6.19E-04	0.013698	Under-represented in disease
Wang et al. (ESRD)	Pathway-level DA	map00561	Glycerolipid metabolism	5.91E-04	0.013698	Over-represented in disease
Wang et al. (ESRD)	Pathway-level DA	map00625	Chloroalkane and chloroalkene degradation	6.54E-04	0.013698	Under-represented in disease
Wang et al. (ESRD)	Pathway-level DA	map00900	Terpenoid backbone biosynthesis	5.94E-04	0.013698	Under-represented in disease
Wang et al. (ESRD)	Pathway-level DA	map00525	Acarbose and validamycin biosynthesis	7.29E-04	0.013906	Under-represented in disease
Wang et al. (ESRD)	Pathway-level DA	map00290	Valine, leucine and isoleucine biosynthesis	0.001071	0.018309	Under-represented in disease
Wang et al. (ESRD)	Pathway-level DA	map00363	Bisphenol degradation	0.001315	0.019305	Over-represented in disease
Wang et al. (ESRD)	Pathway-level DA	map00565	Ether lipid metabolism	0.001323	0.019305	Over-represented in disease
Wang et al. (ESRD)	Pathway-level DA	map00220	Arginine biosynthesis	0.001624	0.022375	Under-represented in disease
Wang et al. (ESRD)	Pathway-level DA	map00380	Tryptophan metabolism	0.001965	0.026335	Over-represented in disease
Wang et al. (ESRD)	Pathway-level DA	map00195	Photosynthesis	0.00214	0.027162	Under-represented in disease
Wang et al. (ESRD)	Pathway-level DA	map00280	Valine, leucine and isoleucine degradation	0.00219	0.027162	Over-represented in disease
Wang et al. (ESRD)	Pathway-level DA	map00400	Phenylalanine, tyrosine and tryptophan biosynthesis	0.002721	0.030676	Under-represented in disease
Wang et al. (ESRD)	Pathway-level DA	map00190	Oxidative phosphorylation	0.003417	0.036062	Under-represented in disease
Wang et al. (ESRD)	Pathway-level DA	map00760	Nicotinate and nicotinamide metabolism	0.004138	0.039475	Under-represented in disease
Wang et al. (ESRD)	Pathway-level DA	map00254	Aflatoxin biosynthesis	0.004274	0.039999	Over-represented in disease
Wang et al. (ESRD)	Pathway-level DA	map00523	Polyketide sugar unit biosynthesis	0.004913	0.044308	Under-represented in disease
Wang et al. (ESRD)	Pathway-level DA	map00030	Pentose phosphate pathway	0.005153	0.045639	Over-represented in disease
Wang et al. (ESRD)	Pathway-level DA	map00640	Propanoate metabolism	0.005369	0.045914	Over-represented in disease
Wang et al. (ESRD)	Pathway-level DA	map00460	Cyanoamino acid metabolism	0.006887	0.053934	Under-represented in disease
Wang et al. (ESRD)	Pathway-level DA	map00660	C5-Branched dibasic acid metabolism	0.007164	0.054667	Under-represented in disease
Wang et al. (ESRD)	Pathway-level DA	map00680	Methane metabolism	0.008692	0.060723	Over-represented in disease
Wang et al. (ESRD)	Pathway-level DA	map00360	Phenylalanine metabolism	0.010326	0.068292	Over-represented in disease
Wang et al. (ESRD)	Pathway-level DA	map00980	Metabolism of xenobiotics by cytochrome P450	0.010259	0.068292	Over-represented in disease
Wang et al. (ESRD)	Pathway-level DA	map00785	Lipoic acid metabolism	0.011633	0.074935	Over-represented in disease
Wang et al. (ESRD)	Pathway-level DA	map00550	Peptidoglycan biosynthesis	0.012094	0.075929	Under-represented in disease
Wang et al. (ESRD)	Pathway-level DA	map00480	Glutathione metabolism	0.013365	0.07987	Over-represented in disease
Wang et al. (ESRD)	Pathway-level DA	map00520	Amino sugar and nucleotide sugar metabolism	0.014646	0.084471	Over-represented in disease
Wang et al. (ESRD)	Pathway-level DA	map00908	Zeatin biosynthesis	0.015252	0.084982	Under-represented in disease
Wang et al. (ESRD)	Pathway-level DA	map00052	Galactose metabolism	0.017638	0.095094	Over-represented in disease
Wang et al. (ESRD)	Pathway-level DA	map00903	Limonene degradation	0.018196	0.097047	Under-represented in disease
Franzosa et al. (CD)	Pathway-level DA	map00220	Arginine biosynthesis	3.87E-09	4.32E-06	Under-represented in disease
Franzosa et al. (CD)	Pathway-level DA	map00332	Carbapenem biosynthesis	1.59E-06	4.45E-04	Under-represented in disease
Franzosa et al. (CD)	Pathway-level DA	map00400	Phenylalanine, tyrosine and tryptophan biosynthesis	3.41E-06	7.60E-04	Under-represented in disease
Franzosa et al. (CD)	Pathway-level DA	map00290	Valine, leucine and isoleucine biosynthesis	1.34E-05	0.002486	Under-represented in disease
Franzosa et al. (CD)	Pathway-level DA	map00340	Histidine metabolism	7.88E-05	0.007703	Under-represented in disease
Franzosa et al. (CD)	Pathway-level DA	map00030	Pentose phosphate pathway	1.03E-04	0.008693	Over-represented in disease
Franzosa et al. (CD)	Pathway-level DA	map00520	Amino sugar and nucleotide sugar metabolism	0.001065	0.042465	Over-represented in disease
Franzosa et al. (CD)	Pathway-level DA	map00052	Galactose metabolism	0.001401	0.052111	Over-represented in disease
Franzosa et al. (CD)	Pathway-level DA	map00254	Aflatoxin biosynthesis	0.001929	0.061446	Over-represented in disease
Franzosa et al. (CD)	Pathway-level DA	map00660	C5-Branched dibasic acid metabolism	0.001771	0.061446	Under-represented in disease
Franzosa et al. (CD)	Pathway-level DA	map00730	Thiamine metabolism	0.001862	0.061446	Under-represented in disease
Franzosa et al. (CD)	Pathway-level DA	map00480	Glutathione metabolism	0.002442	0.064888	Over-represented in disease
Franzosa et al. (CD)	Pathway-level DA	map00950	Isoquinoline alkaloid biosynthesis	0.002879	0.074716	Over-represented in disease
Franzosa et al. (UC)	Pathway-level DA	map00625	Chloroalkane and chloroalkene degradation	5.59E-06	0.002424	Under-represented in disease
Franzosa et al. (UC)	Pathway-level DA	map00310	Lysine degradation	8.58E-05	0.011419	Over-represented in disease
Franzosa et al. (UC)	Pathway-level DA	map00999	Biosynthesis of various plant secondary metabolites	1.72E-04	0.014893	Under-represented in disease
Franzosa et al. (UC)	Pathway-level DA	map00400	Phenylalanine, tyrosine and tryptophan biosynthesis	2.11E-04	0.015238	Under-represented in disease
Franzosa et al. (UC)	Pathway-level DA	map00270	Cysteine and methionine metabolism	3.39E-04	0.021011	Under-represented in disease
Franzosa et al. (UC)	Pathway-level DA	map00450	Selenocompound metabolism	4.75E-04	0.021804	Under-represented in disease
Franzosa et al. (UC)	Pathway-level DA	map00785	Lipoic acid metabolism	4.77E-04	0.021804	Over-represented in disease
Franzosa et al. (UC)	Pathway-level DA	map00290	Valine, leucine and isoleucine biosynthesis	7.48E-04	0.029527	Under-represented in disease
Franzosa et al. (UC)	Pathway-level DA	map00195	Photosynthesis	8.95E-04	0.032357	Under-represented in disease
Franzosa et al. (UC)	Pathway-level DA	map00190	Oxidative phosphorylation	0.001223	0.040829	Under-represented in disease
Franzosa et al. (UC)	Pathway-level DA	map00540	Lipopolysaccharide biosynthesis	0.001463	0.045522	Over-represented in disease
Franzosa et al. (UC)	Pathway-level DA	map00430	Taurine and hypotaurine metabolism	0.001528	0.045747	Over-represented in disease
Franzosa et al. (UC)	Pathway-level DA	map00440	Phosphonate and phosphinate metabolism	0.00206	0.049871	Over-represented in disease
Franzosa et al. (UC)	Pathway-level DA	map00480	Glutathione metabolism	0.002069	0.049871	Over-represented in disease
Franzosa et al. (UC)	Pathway-level DA	map00730	Thiamine metabolism	0.002126	0.049871	Under-represented in disease
Franzosa et al. (UC)	Pathway-level DA	map00650	Butanoate metabolism	0.002634	0.060167	Over-represented in disease
Franzosa et al. (UC)	Pathway-level DA	map00592	alpha-Linolenic acid metabolism	0.002773	0.061707	Over-represented in disease
Franzosa et al. (UC)	Pathway-level DA	map00680	Methane metabolism	0.003179	0.064171	Over-represented in disease
Franzosa et al. (UC)	Pathway-level DA	map00020	Citrate cycle (TCA cycle)	0.003327	0.065627	Over-represented in disease
Franzosa et al. (UC)	Pathway-level DA	map00030	Pentose phosphate pathway	0.004094	0.071889	Over-represented in disease
Franzosa et al. (UC)	Pathway-level DA	map00053	Ascorbate and aldarate metabolism	0.004141	0.071889	Over-represented in disease
Franzosa et al. (UC)	Pathway-level DA	map00332	Carbapenem biosynthesis	0.004228	0.071961	Under-represented in disease
Franzosa et al. (UC)	Pathway-level DA	map00220	Arginine biosynthesis	0.004753	0.075299	Under-represented in disease
Franzosa et al. (UC)	Pathway-level DA	map01053	Biosynthesis of siderophore group nonribosomal peptides	0.004771	0.075299	Over-represented in disease
Franzosa et al. (UC)	Pathway-level DA	map00401	Novobiocin biosynthesis	0.006934	0.096367	Under-represented in disease
Franzosa et al. (UC)	Pathway-level DA	map00770	Pantothenate and CoA biosynthesis	0.006869	0.096367	Under-represented in disease
Jeffery et al. (IBS)	Pathway-level DA	map00470	D-Amino acid metabolism	8.13E-05	0.002804	Under-represented in disease
Jeffery et al. (IBS)	Pathway-level DA	map00550	Peptidoglycan biosynthesis	1.39E-04	0.002804	Under-represented in disease

Jeffery et al. (IBS)	Pathway-level DA	map00900	Terpenoid backbone biosynthesis	5.14E-05	0.002804	Under-represented in disease
Jeffery et al. (IBS)	Pathway-level DA	map00908	Zeatin biosynthesis	1.17E-04	0.002804	Under-represented in disease
Jeffery et al. (IBS)	Pathway-level DA	map00970	Aminoacyl-tRNA biosynthesis	9.24E-05	0.002804	Under-represented in disease
Jeffery et al. (IBS)	Pathway-level DA	map00983	Drug metabolism - other enzymes	9.48E-05	0.002804	Under-represented in disease
Jeffery et al. (IBS)	Pathway-level DA	map00561	Glycerolipid metabolism	1.76E-04	0.003047	Over-represented in disease
Jeffery et al. (IBS)	Pathway-level DA	map00010	Glycolysis / Gluconeogenesis	2.27E-04	0.00343	Under-represented in disease
Jeffery et al. (IBS)	Pathway-level DA	map00240	Pyrimidine metabolism	2.61E-04	0.003507	Under-represented in disease
Jeffery et al. (IBS)	Pathway-level DA	map00620	Pyruvate metabolism	6.19E-04	0.007487	Over-represented in disease
Jeffery et al. (IBS)	Pathway-level DA	map00052	Galactose metabolism	7.26E-04	0.007987	Over-represented in disease
Jeffery et al. (IBS)	Pathway-level DA	map00640	Propanoate metabolism	7.92E-04	0.007987	Over-represented in disease
Jeffery et al. (IBS)	Pathway-level DA	map00254	Aflatoxin biosynthesis	9.56E-04	0.008896	Over-represented in disease
Jeffery et al. (IBS)	Pathway-level DA	map00520	Amino sugar and nucleotide sugar metabolism	0.001148	0.009275	Over-represented in disease
Jeffery et al. (IBS)	Pathway-level DA	map00650	Butanoate metabolism	0.00115	0.009275	Over-represented in disease
Jeffery et al. (IBS)	Pathway-level DA	map00910	Nitrogen metabolism	0.002467	0.018653	Over-represented in disease
Jeffery et al. (IBS)	Pathway-level DA	map00051	Fructose and mannose metabolism	0.003	0.021354	Over-represented in disease
Jeffery et al. (IBS)	Pathway-level DA	map00140	Steroid hormone biosynthesis	0.004404	0.029147	Over-represented in disease
Jeffery et al. (IBS)	Pathway-level DA	map00600	Sphingolipid metabolism	0.004577	0.029147	Over-represented in disease
Jeffery et al. (IBS)	Pathway-level DA	map00627	Aminobenzoate degradation	0.005181	0.031343	Over-represented in disease
Jeffery et al. (IBS)	Pathway-level DA	map00630	Glyoxylate and dicarboxylate metabolism	0.007873	0.045366	Over-represented in disease
Jeffery et al. (IBS)	Pathway-level DA	map00770	Pantothenate and CoA biosynthesis	0.008449	0.046469	Under-represented in disease
Jeffery et al. (IBS)	Pathway-level DA	map00332	Carbapenem biosynthesis	0.009367	0.049279	Under-represented in disease
Jeffery et al. (IBS)	Pathway-level DA	map00071	Fatty acid degradation	0.01074	0.052993	Over-represented in disease
Jeffery et al. (IBS)	Pathway-level DA	map00670	One carbon pool by folate	0.010949	0.052993	Under-represented in disease
Jeffery et al. (IBS)	Pathway-level DA	map00710	Carbon fixation in photosynthetic organisms	0.013107	0.061	Under-represented in disease
Jeffery et al. (IBS)	Pathway-level DA	map00280	Valine, leucine and isoleucine degradation	0.014809	0.063997	Over-represented in disease
Jeffery et al. (IBS)	Pathway-level DA	map00511	Other glycan degradation	0.014519	0.063997	Over-represented in disease
Jeffery et al. (IBS)	Pathway-level DA	map00780	Biotin metabolism	0.016594	0.069238	Over-represented in disease
Jeffery et al. (IBS)	Pathway-level DA	map00531	Glycosaminoglycan degradation	0.018977	0.075413	Over-represented in disease
Jeffery et al. (IBS)	Pathway-level DA	map00760	Nicotinate and nicotinamide metabolism	0.019321	0.075413	Under-represented in disease
Jeffery et al. (IBS)	Pathway-level DA	map00030	Pentose phosphate pathway	0.022655	0.085346	Over-represented in disease
Jeffery et al. (IBS)	Pathway-level DA	map00053	Ascorbate and aldarate metabolism	0.024009	0.085346	Over-represented in disease
Jeffery et al. (IBS)	Pathway-level DA	map00720	Carbon fixation pathways in prokaryotes	0.023691	0.085346	Under-represented in disease
Jeffery et al. (IBS)	Pathway-level DA	map01053	Biosynthesis of siderophore group nonribosomal peptides	0.024687	0.085346	Over-represented in disease
Jeffery et al. (IBS)	Pathway-level DA	map00380	Tryptophan metabolism	0.026153	0.087903	Over-represented in disease
Jeffery et al. (IBS)	Pathway-level DA	map00195	Photosynthesis	0.027123	0.088699	Under-represented in disease
Jeffery et al. (IBS)	Pathway-level DA	map00230	Purine metabolism	0.028699	0.08998	Under-represented in disease
Jeffery et al. (IBS)	Pathway-level DA	map00310	Lysine degradation	0.029002	0.08998	Over-represented in disease
Jeffery et al. (IBS)	Pathway-level DA	map00190	Oxidative phosphorylation	0.029755	0.090008	Under-represented in disease
Jeffery et al. (IBS)	Pathway-level DA	map00604	Glycosphingolipid biosynthesis - ganglio series	0.030845	0.091031	Over-represented in disease
Wang et al. (ESRD)	ORA-ECs	map00730	Thiamine metabolism	4.08E-04	0.022413	Under-represented in disease
Franzosa et al. (CD)	ORA-ECs	map00230	Purine metabolism	0.001781	0.099747	Over-represented in disease
Franzosa et al. (CD)	ORA-ECs	map00220	Arginine biosynthesis	8.11E-05	0.00454	Under-represented in disease
Franzosa et al. (CD)	ORA-ECs	map00340	Histidine metabolism	1.97E-04	0.005519	Under-represented in disease
Franzosa et al. (UC)	ORA-ECs	map00540	Lipopolysaccharide biosynthesis	5.62E-06	3.15E-04	Over-represented in disease
Franzosa et al. (UC)	ORA-ECs	map00785	Lipoic acid metabolism	0.001267	0.035478	Over-represented in disease
Franzosa et al. (UC)	ORA-ECs	map00480	Glutathione metabolism	0.002465	0.046014	Over-represented in disease
Franzosa et al. (UC)	ORA-ECs	map00400	Phenylalanine, tyrosine and tryptophan biosynthesis	4.34E-04	0.024307	Under-represented in disease
Franzosa et al. (UC)	ORA-ECs	map00340	Histidine metabolism	0.001357	0.03799	Under-represented in disease
Franzosa et al. (UC)	ORA-ECs	map00540	Lipopolysaccharide biosynthesis	1.84E-04	0.010308	Associated with disease
Franzosa et al. (CD)	ORA-Metabolites	map04978	Mineral absorption	2.27E-04	0.014999	Over-represented in disease
Franzosa et al. (CD)	ORA-Metabolites	map00120	Primary bile acid biosynthesis	0.0012	0.039608	Over-represented in disease
Franzosa et al. (CD)	ORA-Metabolites	map04974	Protein digestion and absorption	0.002147	0.047235	Over-represented in disease
Jeffery et al. (IBS)	ORA-Metabolites	map04974	Protein digestion and absorption	1.24E-05	7.09E-04	Over-represented in disease
Jeffery et al. (IBS)	ORA-Metabolites	map00970	Aminoacyl-tRNA biosynthesis	1.12E-04	0.003182	Over-represented in disease
Jeffery et al. (IBS)	ORA-Metabolites	map00270	Cysteine and methionine metabolism	1.98E-04	0.003761	Over-represented in disease
Jeffery et al. (IBS)	ORA-Metabolites	map04978	Mineral absorption	3.43E-04	0.004885	Over-represented in disease
Jeffery et al. (IBS)	ORA-Metabolites	map00997	Biosynthesis of various other secondary metabolites	0.002531	0.026665	Over-represented in disease
Jeffery et al. (IBS)	ORA-Metabolites	map00470	D-Amino acid metabolism	0.003275	0.026665	Over-represented in disease
Jeffery et al. (IBS)	ORA-Metabolites	map05230	Central carbon metabolism in cancer	0.003275	0.026665	Over-represented in disease
Jeffery et al. (IBS)	ORA-Metabolites	map01230	Biosynthesis of amino acids	0.005957	0.042447	Over-represented in disease
Jeffery et al. (IBS)	ORA-Metabolites	map00260	Glycine, serine and threonine metabolism	0.015949	0.095025	Over-represented in disease
Jeffery et al. (IBS)	ORA-Metabolites	map01210	2-Oxocarboxylic acid metabolism	0.016671	0.095025	Over-represented in disease
Jeffery et al. (IBS)	ORA-Metabolites	map04974	Protein digestion and absorption	7.70E-04	0.043874	Associated with disease
Jeffery et al. (IBS)	ORA-Metabolites	map00970	Aminoacyl-tRNA biosynthesis	0.002521	0.071859	Associated with disease

References

1. Pittayanon, R. *et al.* Gut Microbiota in Patients With Irritable Bowel Syndrome—A Systematic Review. *Gastroenterology* **157**, 97–108 (2019).

2. Mars, R. A. T. *et al.* Longitudinal Multi-omics Reveals Subset-Specific Mechanisms Underlying Irritable Bowel Syndrome. *Cell* **182**, 1460-1473.e17 (2020).

3. Jacobs, J. P. *et al.* Multi-omics profiles of the intestinal microbiome in irritable bowel syndrome and its bowel habit subtypes. *Microbiome* **11**, 1–18 (2023).

4. Jeffery, I. B. *et al.* Differences in Fecal Microbiomes and Metabolomes of People With vs Without Irritable Bowel Syndrome and Bile Acid Malabsorption. *Gastroenterology* **158**, 1016-1028.e8 (2020).

5. Edogawa, S. *et al.* Serine proteases as luminal mediators of intestinal barrier dysfunction and symptom severity in irritable bowel syndrome. *Gut* **69**, 62–73 (2021).

6. Steck, N., Mueller, K., Schemann, M. & Haller, D. Bacterial proteases in IBD and IBS. *Postgrad. Med. J.* **89**, 25–33 (2013).

7. Nüse, B., Holland, T., Rauh, M., Gerlach, R. G. & Mattner, J. L-arginine metabolism as pivotal interface of mutual host–microbe interactions in the gut. *Gut Microbes* **15**, (2023).

8. Berstad, A., Raa, J. & Valeur, J. Tryptophan: 'Essential' for the pathogenesis of irritable bowel syndrome? *Scand. J. Gastroenterol.* **49**, 1493–1498 (2014).

9. Plantinga, A. M. *et al.* Exploration of associations among dietary tryptophan, microbiome composition and function, and symptom severity in irritable bowel syndrome. *Neurogastroenterology and Motility* **35**, 1–11 (2023).

10. Bhattarai, Y. *et al.* Gut Microbiota-Produced Tryptamine Activates an Epithelial G-Protein-Coupled Receptor to Increase Colonic Secretion. *Cell Host Microbe* **23**, 775-785.e5 (2018).

11. Xiao, L., Liu, Q., Luo, M. & Xiong, L. Gut Microbiota-Derived Metabolites in Irritable Bowel Syndrome. *Front. Cell. Infect. Microbiol.* **11**, 1–11 (2021).

12. Mars, R. A. T. *et al.* Longitudinal Multi-omics Reveals Subset-Specific Mechanisms Underlying Irritable Bowel Syndrome. *Cell* **182**, 1460-1473.e17 (2020).

13. Chellappa, K. *et al.* NAD precursors cycle between host tissues and the gut microbiome. *Cell Metab.* **34**, 1947-1959.e5 (2022).

14. Jacobs, J. P. *et al.* Multi-omics profiles of the intestinal microbiome in irritable bowel syndrome and its bowel habit subtypes. *Microbiome* **11**, 1–18 (2023).

15. Kanehisa, M. & Goto, S. KEGG: kyoto encyclopedia of genes and genomes. *Nucleic Acids Res.* **28**, 27–30 (2000).

16. Huss, M. & Holme, P. Currency and commodity metabolites: Their identification and relation to the modularity of metabolic networks. *IET Syst. Biol.* **1**, 280–285 (2007).

17. Franzosa, E. A. *et al.* Gut microbiome structure and metabolic activity in inflammatory bowel disease. *Nat. Microbiol.* **4**, 293–305 (2019).

18. Jeffery, I. B. *et al.* Differences in Fecal Microbiomes and Metabolomes of People With vs Without Irritable Bowel Syndrome and Bile Acid Malabsorption. *Gastroenterology* **158**, 1016-1028.e8 (2020).

19. Wang, X. *et al.* Aberrant gut microbiota alters host metabolome and impacts renal failure in humans and rodents. *Gut* **69**, 2131–2142 (2020).

20. Beghini, F. *et al.* Integrating taxonomic, functional, and strain-level profiling of diverse microbial communities with biobakery 3. *Elife* **10**, (2021).

21. Chen, S., Zhou, Y., Chen, Y. & Gu, J. fastp: an ultra-fast all-in-one FASTQ preprocessor. *Bioinformatics* **34**, i884–i890 (2018).

22. Manor, O. & Borenstein, E. MUSiCC: a marker genes based framework for metagenomic normalization and accurate profiling of gene abundances in the microbiome. *Genome Biol.* **16**, 53 (2015).

23. Chong, J. *et al.* MetaboAnalyst 4.0: towards more transparent and integrative metabolomics analysis | Nucleic Acids Research | Oxford Academic. *Nucleic Acids Res.* **46**, W486–W494 (2018).

24. van den Berg, R. A., Hoefsloot, H. C., Westerhuis, J. A., Smilde, A. K. & van der Werf, M. J. Centering, scaling, and transformations: improving the biological information content of metabolomics data. *BMC Genomics* **7**, 142 (2006).

25. Mallick, H. *et al.* Multivariable association discovery in population-scale meta-omics studies. *PLoS Comput. Biol.* **17**, 1–27 (2021).

26. May, A. *et al.* metaModules identifies key functional subnetworks in microbiome-related disease. *Bioinformatics* **32**, 1678–1685 (2016).

27. Dittrich, M. T., Klau, G. W., Rosenwald, A., Dandekar, T. & Müller, T. Identifying functional modules in protein-protein interaction networks: An integrated exact approach. *Bioinformatics* **24**, (2008).

28. Beisser, D., Klau, G. W., Dandekar, T., Müller, T. & Dittrich, M. T. BioNet: An R-Package for the functional analysis of biological networks. *Bioinformatics* **26**, 1129–1130 (2010).

29. Csardi, G. & Nepusz, T. The igraph software package for complex network research. *InterJournal Complex Systems* 1–9 (2006).

30. Manor, O. & Borenstein, E. Revised computational metagenomic processing uncovers hidden and biologically meaningful functional variation in the human microbiome. *Microbiome* **5**, 1–11 (2017).

31. Gustavsen, J. A., Pai, S., Isserlin, R., Demchak, B. & Pico, A. R. RCy3: Network biology using Cytoscape from within R. *F1000Research 2019 8:1774* **8**, 1774 (2019).

32. Smoot, M. E., Ono, K., Ruscheinski, J., Wang, P.-L. & Ideker, T. Systems biology Cytoscape 2.8: new features for data integration and network visualization. *BIOINFORMATICS APPLICATIONS NOTE* **27**, 431–432 (2011).

33. Pounds, S. & Morris, S. W. Estimating the occurrence of false positives and false negatives in microarray studies by approximating and partitioning the empirical distribution of p-values. *Bioinformatics* **19**, 1236–1242 (2003).
